# Correlating gene expression levels with transcription factor binding sites facilitates identification of key transcription factors from transcriptome data

**DOI:** 10.3389/fgene.2024.1511456

**Published:** 2024-11-29

**Authors:** Tinghua Huang, Siqi Niu, Fanghong Zhang, Binyu Wang, Jianwu Wang, Guoping Liu, Min Yao

**Affiliations:** ^1^ College of Animal Science and Technology, Yangtze University, Jingzhou, China; ^2^ College of Agriculture, Yangtze University, Jingzhou, China

**Keywords:** transcription factor, transcriptome data, correlation analysis, Kendall’s tau, Jinzer, Flaver

## Abstract

Identification of key transcription factors from transcriptome data by correlating gene expression levels with transcription factor binding sites is important for transcriptome data analysis. In a typical scenario, we always set a threshold to filter the top ranked differentially expressed genes and top ranked transcription factor binding sites. However, correlation analysis of filtered data can often result in spurious correlations. In this study, we tested four methods for creating the gene expression inputs (ranked gene list) in the correlation analysis: star coordinate map transformation (START), expression differential score (ED), preferential expression measure (PEM), and the specificity measure (SPM). Then, Kendall’s tau correlation statistical algorithms implementing the standard (STD), LINEAR, MIX-LINEAR, DENSITY-CURVE, and MIXED-DENSITY-CURVE weighting methods were used to identify key transcription factors. ED was identified as the optimal method for creating a ranked gene list from filtered expression data, which can address the “unable to detect negative correlation” fallacy presented by other methods. The MIXED-DENSITY-CURVE was the most sensitive for identifying transcription factors from the gene set and list in which only the top proportion was correlated. Ultimately, 644 transcription factor candidates were identified from the transcriptome data of 1,206 cell lines, six of which were validated by wet lab experiments. The Jinzer and Flaver software implementing these methods can be obtained from http://www.thua45/cn/flaver under a free academic license.

## 1 Introduction

We started the analysis by creating the gene expression inputs (ranked gene list) for the significance of differential expression in a specific cell line or tissue sample. Identifying cell-specific expressed genes and the key transcription factors (TF) that regulate these genes from transcriptome data continue to pose challenges. At present, there are five representative state-of-the-art indexes for estimating and identifying the significance of sample-specific gene expression. Let x_i_ be the expression level of gene x in sample i, s_i_ summarize the expression levels of all genes in sample i, and n be the number of samples, which are calculated as follows:

The sample specificity expressed index τ defined as formula 1 was proposed by Yanai et al. (2005).

$$\tau =\frac{\sum_{i=1}^{n}\left[1-x_{i}/\left(\max_{1\leq i\leq n}x_{i}\right)\right]}{n-1}$$ (1)

As presented by Yu et al. (2006), the extent of tissue or cell line-specific expression is defined for each gene as EE (expression enrichment), and was calculated according to formula 2.

$$\text{EE}=x_{i}/\left[\left(\sum_{i=1}^{n}x_{i}\right)*\left(s_{i}/\sum_{i=1}^{n}s_{i}\right)\right]$$ (2)

The H_g_ score measures the sample-specificity with entropy (Schug et al., 2005), calculated as shown in formula 3.

$$H_{g}=\sum_{i=1}^{n}-\left[\frac{x_{i}}{\sum_{i=1}^{n}x_{i}}*\log_{2}\left(\frac{x_{i}}{\sum_{i=1}^{n}x_{i}}\right)\right]$$ (3)

The specificity measure (SPM) from the TiSGeD database (Xiao et al., 2010) was calculated using formula 4.

$$\text{SPM}=\frac{x_{i}^{2}}{\sum_{i=1}^{n}x_{i}^{2}}$$ (4)

The preferential expression measure (PEM), which estimates how different the expression of the gene is relative to an expected expression (Huminiecki et al., 2003), can be calculated using formula 5.

$$\text{PEM}=\log_{10}\left[x_{i}/\left(\frac{s_{i}*\sum_{i=1}^{n}x_{i}}{\sum_{i=1}^{n}s_{i}}\right)\right]$$ (5)

In a previous study, Kryuchkova-Mostacci et al. conducted a detailed evaluation and review of these indexes (Kryuchkova-Mostacci and Robinson-Rechavi, 2017). All these indexes focus on identifying genes that are specifically expressed in a certain sample or cell line, particularly those expressed dominantly.

A variety of factors including transcription factor binding, DNA accessibility changes, and sequence specificity determined the transcriptional levels of genes. Current transcription factor mining methods test the significance of a set of genes regulated by a transcription factor (gene set) in a list of differentially expressed genes (gene list) identified by gene expression profiling. Representative software includes GOStats (Falcon and Gentleman, 2007), ClusterProfiler (Yu et al., 2012), GSEA (Subramanian et al., 2005), WebGestalt (Wang et al., 2017), and Flaver (Yao et al., 2024), among others (Maleki et al., 2020). GOStats software and its alternatives implement Fisher’s exact test (Yao et al., 2024; Keenan et al., 2019; Magnusson and Lubovac-Pilav, 2021; Lachmann et al., 2010), GSEA software implements the Kolmogorov Smirnov statistics (Subramanian et al., 2005; Maleki et al., 2020), and ClusterProfiler and WebGestalt implement both statistics (Yu et al., 2012; Wang et al., 2017). Among these, GOStats (Falcon and Gentleman, 2007) uses gene sets and gene lists without rank attributes, whereas ClusterProfiler and GSEA software use gene sets without rank attributes and gene lists with rank attributes. The HOMER (Heinz et al., 2010) and MEME (Bailey et al., 2009) software implemented comprehensive novel motif discovery and motif scanning algorithms that were designed for regulatory element analysis in genomics applications. ARACNE (Margolin et al., 2006) was an algorithm for the reconstruction of gene regulatory networks in a mammalian cellular context, and its partner VIPER (Alvarez et al., 2016) which implemented the aREA algorithm, testing for a global shift in the positions of each regulon genes when projected on the rank-sorted gene expression signature which is similar to GSEA’s concepts. The recently developed Flaver software (Yao et al., 2024) employs weighted Kendall’s tau rank correlation statistics, which support both ranked gene lists and ranked gene set inputs. The rank attributes of the genes in the gene list can be a measure of the degree of gene expression difference. The rank attributes of genes in the gene set may be the true or false possibilities for transcription factor binding sites.

In this study, a two-step pipeline was developed to analyze transcriptome data. A ranked gene list was created using four different methods: star coordinate map transformation (START), expression differential score (ED), preferential expression measure (PEM), and the specificity measure (SPM). TF-derived gene sets with rank information were created using the Jinzer software with updated promoter sequences and algorithms. Flaver software was developed to identify significant correlations between ranked gene sets and ranked gene lists. Finally, we applied the pipeline to transcriptome data from 1,206 human cell lines and obtained promising results, demonstrating its desirability as a gene set discovery tool.

## 2 Materials and methods

### 2.1 Creation of a ranked gene list from transcriptome data and four indexes

The transcriptome data used in this study were obtained from the Protein Atlas database (Uhlén et al., 2015), including 1,206 human cell lines (quantile normalized). For the calculation of the four indexes, the SPM and PEM scores were specified using formula 4, 5 without modification. The index τ and H_g_ score only estimated the overall sample-specific expression per gene. The EE score was the same as the inverse logarithm form of PEM. These three indexes were not included in this study. The expression differential (ED) score was calculated using formula 6. The star coordinate transformation method is as follows:

$$\text{ED}=x_{i}-\left[\left(\sum_{i=1}^{n}x_{i}\right)*\left(s_{i}/\sum_{i=1}^{n}s_{i}\right)\right]$$ (6)

The star coordinate (START) method is based on parallel coordinates (Heinrich and Weiskopf, 2009) and star coordinates (Kandogan, 2000). This study adopted a similar strategy: first, gene expression data were mapped to a two-dimensional space, and then the degree of differential gene expression was calculated (Supplementary Figure S1). The arrangement order of each coordinate axis was determined by hierarchical clustering method. The START method defines a two-dimensional vector A = [A1, A2, A3, … , An] that starts at the origin (Ox, Oy) and extends along the coordinate axis. Let the initial value of the data points be D = (D1, D2, D3, … , Dn) and set to the lengths of the two-dimensional vectors Ai. The Cartesian coordinates of the two-dimensional vector Ai corresponding to Di are (Di * cos Ɵ, Di * sin Ɵ), where Ɵ is the angle of coordinates. The final Cartesian coordinate value of data point D can be obtained from the sum of Ai, as specified in formula 7:

$$P\left(x,y\right)=\left(O_{x}+\sum_{i=1}^{n}D_{i}∙\cos\theta ,O_{y}+\sum_{i=1}^{n}D_{i}∙\sin\theta \right)$$ (7)

**FIGURE 1:**
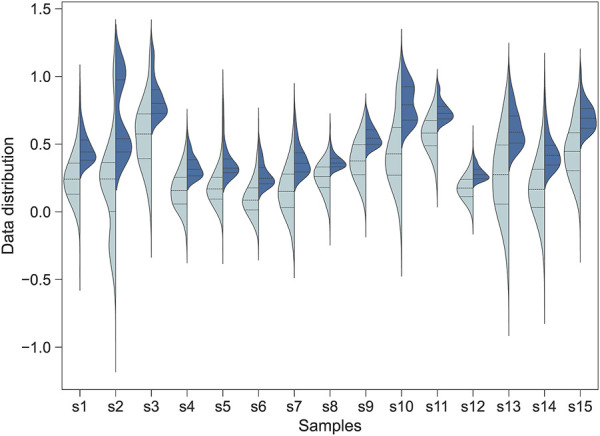
The distribution of the Jindex score for the transcription factor binding sites identified by Jinzer software. Left: A full-spectrum distribution. Right: The partial distribution for Jindex >1.0. The plot shows the distributions for all binding sites of 15 randomly selected transcription factors in the human genome.

The distance from point V, which is the vertical intersection point for point P to the coordinate axis of the target sample to the origin site O is a perfect measure of the degree of gene expression difference (Supplemental document Figure 1A). Let δ be the angle of OP, the length of VO can be calculated by formula 8:

$$\text{VO}=\sqrt[{2}]{P_{x}^{2}*P_{y}^{2}}*\cos\left|\theta -\delta \right|$$ (8)

### 2.2 Development of an improved version of transcription factor binding site prediction software

In our previous study, we developed a genome-wide transcription factor binding site prediction software, Grit (Huang et al., 2022), based on comparative genomics and the mixed Student’s t-test. In this study, we revised the calculation method for the raw binding site score (RS), specified in formula 9, 10. This revised score was denoted as “Jindex”. The reason for this revision is that, according to Markov Chain theory, the relationship between the probabilities of individual parts should be multiplicative rather than additive.

$$\text{RS}=\ln\frac{1}{M_{s}}\sum_{L=1}^{M_{s}}\prod_{k=1}^{w}\frac{q\left(k,L_{k}\right)}{p\left(L_{k}\right)}$$ (9)

$$\text{Jindex}={\mathrm{Max}}_{s}\left\{\ln\sqrt[{w}]{\prod_{k=1}^{w}\frac{q\left(k,L_{k}\right)}{p\left(L_{k}\right)}}\right\};\;1\leq s\leq l-w+1$$ (10)

The Jindex calculation represents the maximum of repeated averaging of log likelihood ratios (LLRs). The averaged LLR indicated the possibility for a motif being present at one particular location in a sequence, where *w* was the width of the motif, *L* denoted the location being considered, *L*_*k*_ was the nucleotide at position *k* within this location, *p(L*_*k*_ *)* is the background probability of observing nucleotide *L*_*k*_ estimated from the frequency of *L*_*k*_ in that sequence, and *q(k, L*_*k*_ *)* is the probability of observing nucleotide *L*_*k*_ estimated from the frequency of the *Kth* location in the motif. The Jindex for a motif present in a sequence with length *l* was the maximum of the average of LLRs taken over all locations of *s*, where *M*_*s*_ was the number of locations in the sequence calculated as *l*–*w* + 1. Jindex value >1.0 indicated that the probability of observing the motif in the sequence is higher than the background probability.

A total of 1,575 position weight matrixs (PWMs) for transcription factors were obtained from public sources (Vorontsov et al., 2024; Rauluseviciute et al., 2024). The newest promoter sequence set (550-bp set) was obtained from Jinzer’s website (Yao et al., 2024). Jinzer software was run with PWMs and the promoter sequence set identified candidate transcription factor binding site (TFBS) with Jindex ≥1.0. A gene set was created by assigning target genes to TFs if the gene had at least one TFBS. Each TF–target gene pair was assigned a rank value, which was the Jindex of Jinzer’s output. However, it should be aware that the analysis presented in the manuscript focused on the −1 ∼ −500bp upstream sequence which will lose distal transcription factor binding site located out of this region.

### 2.3 Identification of key transcription factors from transcriptome data

Flaver software (Yao et al., 2024), which implements weighted Kendall’s tau statistics, was used to identify the key transcription factors within each cell line. This method allows for testing the significance of the correlation between the rank orders of genes in the gene set and their corresponding rank orders within the gene list. Let Si and Li, i = 1, … , n be the ranks of the gene set or list, respectively. Furthermore, let (i, Ri), i = 1, … , n be paired ranks, where Ri is a rank entity of L, whose corresponding S has rank i among Sj, j = 1, … , n. Kendall’s tau has the form of formula 11 and the limiting distribution (LD), following the U-statistics of formula 12 approximated to N (0, 1). This method was implemented in Faver software as an STD (-w 0 option):

$$\tau =2/n\left(n-1\right)∙\sum_{i>j}^{n}\text{sgn}\left(i-j\right)\text{sgn}\left(R_{i}-R_{j}\right)$$ (11)

$$\text{LD}=3\sqrt{\frac{n\left(n-1\right)}{2\left(2n+5\right)}}∙\tau$$ (12)

Shieh (1998) discovered a weighted version of the rank correlation, the weighted Kendall’s tau, which has the form of formula 13:

$$\tau_{w}=2/\left[{\left(\sum_{i}^{n}v_{i}\right)}^{2}-\sum_{i}^{n}v_{i}^{2}\right]∙\sum_{i>j}^{n}v_{i}v_{j}\text{sgn}\left(i-j\right)\text{sgn}\left(R_{i}-R_{j}\right)$$ (13)

The sgn(x) = −1, 0 or 1, if x <, = or >0, and v_i_ represents the weighting function bounded to [1, n] and ranges from (0, 1). The limiting distribution (LD) can be derived using formula 14:

$${\text{LD}}_{w}=\sqrt{n}\tau_{w}\frac{3\lim_{n\to \infty }n^{-1}\sum_{i}^{n}v_{i}}{2\sqrt{\lim_{n\to \infty }n^{-1}\sum_{i}^{n}v_{i}^{2}}}$$ (14)

Where when n→∞, LD approximated to N(0, 1).

Four weighting functions were developed in this study, ranging from 0 to 1. The first two weighting functions were either based on the geometric mean of the gene ranks in gene set i_s_ and gene list (i_l_) or separately (formulas 15, 16) to determine how the genes in the top-ranked TF–target gene pairs in the gene set correlated with the top-ranked differentially expressed gene in the gene list. The remaining two weighting functions were either based on the geometric mean of the density of genes in gene set d_s_ and gene list (d_l_) or separately (formulas 17, 18). The source code was deposited in GitHub and is available under a free academic license (https://github.com/thua45/flaver). The v_1_ to v_4_ methods were implemented in Flaver software as MIX-LINEAR, LINEAR, MIXED-DENSITY-CURVE, and DENSITY-CURVE, and can be specified by options w 1, 2, 7, and 8, respectively.

$$v_{1}=\sqrt{\left(1-\frac{\left|i_{s}\left|-0.5\right.\right.}{n}\right)∙\left(1-\frac{\left|i_{l}\left|-0.5\right.\right.}{n}\right)}$$ (15)

$$v_{2}=1-\left(\left|i_{x}\right|-0.5\right)/n,x=s\text{ or }l$$ (16)

$$v_{3}=\sqrt{\left(1-\frac{d_{s}}{\max\left(d_{s}\right)}\right)∙\left(1-\frac{d_{l}}{\max\left(d_{l}\right)}\right)}$$ (17)

$$v_{4}=1-\left[d_{x}/\max\left(d_{x}\right)\right],x=s\text{ or }l$$ (18)

### 2.4 Cell culture and flow cytometry assay

HL-60, MOLM-13, OCI-AML-3, and U-937 cell lines were purchased from Procell (Wuhan, China). HL-60, MOLM-13 and U937 cells were cultured in an RPMI 1640 medium supplemented with 10% FBS, 1% glutamine and 1% penicillin–streptomycin (ThermoFisher Scientific, Dreieich, Germany). OCI-AML-3 cells were cultured in a complete medium consisting of 84% IMDM, 15% FBS, and 1% penicillin streptomycin (ThermoFisher Scientific, Dreieich, Germany). All these suspension cell lines were maintained in culture at a density below 1 × 10^6^ cells/mL and were used for seeding in 6-well plates with 1 × 10^6^ cells per well. All cell lines were grown in a humidified air incubator at 37°C containing 5% CO_2_. The cells were treated with shRNA lentiviral vectors targeting ZNF460, SPI1, SPIB, ZNF384, ZNF784, and BATF3 (detailed information available in Supplemental document) following manufacturer’s instructions, and cell proliferation was measured using CellTrace™ CFSE Cell Proliferation Kit (ThermoFisher Scientific, Cat NO. C34554).

## 3 Results

### 3.1 Overview of ranked gene sets

The Jinzer run took 2 h on a 8-core Dell desktop computer and identified a total of 5.91 million significant TFBS (Jindex ≥1.0). The number of target genes found in at least one TFBS for the PWMs varied from 1,548 to 7,150. TGIF1 and OVOL2 had the highest and lowest number of target genes, respectively. Compared to the 11,539 human H3K27ac Chip-Seq datasets collected from the Chip-Atlas database (Zou et al., 2024), 60.4% of the TFBS candidates were supported by experimental evidence. For example, NFKB1 transcription has 3,879 candidate target genes with at least one TFBS identified by Jinzer. Among these, 68.3% of the targets had a Chip-Seq pick covering the TFBS, in line with our prediction. The Jinzer gene set file was created by assigning a RANK score to each TFtarget gene pair, which was assigned from the Jindex value.

The distribution of Jindex for the transcription factor binding sites identified by Jinzer is shown in Figure 1. The results indicated that the Jindex values of the binding sites for the TF-target gene pairs were approximately normally distributed (Figure 1A). Because binding sites with higher Jindex values are more likely to be true binding sites, we set a threshold value for Jindex in practice; that is, binding sites with Jindex scores greater than the threshold value were considered candidate sites (Figure 1B). These candidate loci can be converted into a ranked gene set and used as an input file for Flaver, denoted as “Jinzer set” in this study.

### 3.2 Creation of ranked gene list

**FIGURE 2:**
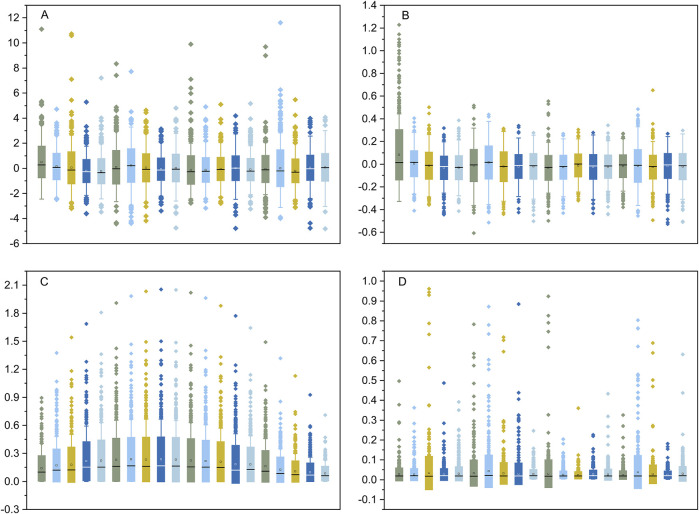
The distribution of the rank values of the gene list created by the DE, PEM, START, and SPM indexes. The DE and PEM indexes shown in plot **(A, B)**, respectively, were approximately normal distributed. The START and SPM indexes shown in plot **(C, D)** were severely skewed. The boxes show the 25% and 75% percentile, and the whiskers show the 5% and 95% percentile.

Transcriptome data from 1,206 human cell lines were transformed into ranked cell-specific gene lists using the four indexes described in the Materials and Methods section. The ranks of the ED (Figure 2A) and PEM (Figure 2B) indexes were similar and approximately normally distributed. The major difference between the distributions of the ranks of the ED and PEM indexes was that the standard deviation of the ED index was significantly higher than that of the PEM index. The results of the START (Figure 2C) were unique, informative, and interesting. The gene list created using the SPM index was severely skewed and distributed (Figure 2D). We performed a gene search according to the gene’s Gene Ontology (GO) annotation and found an average of 22.3% overlap between the GO gene sets annotated with sample-specific functions and the cell-specific gene lists identified by the four indexes. This suggested that many genes with known sample-related functions were also dominantly or recessively expressed in the corresponding transcriptome data and *vice versa*. The gene lists created by the four indexes, named the CELLINE list, were used as inputs for the Flaver software.

### 3.3 Generate and test simulated dataset

R (version 4.2) was used to generate four synthetic gene sets and gene list data to simulate the gene expression and transcription factor binding site data to the real data distribution properties. The gene set and gene list in simulated dataset 1 were normally distributed random data with covariance (R) range of [-1, 1] (Figure 2A shows the simulated data with R = 0.8). For simulated dataset 2, a threshold condition of RANK >0 was set for both the gene set and gene list, in addition to simulated dataset 1, which was used to simulate filtering high-scoring binding sites and upregulated genes when analyzing transcriptome data (Figure 2B shows the simulated data with R = 0.8). The gene set and gene list in simulation dataset 3 were also normally distributed random data with a covariance range in [-1, 1]. However, in contrast to simulation dataset 2, only a filter condition of RANK gene set greater than zero was set, and the gene list had a full-spectrum distribution (Figure 2C shows the simulation data with R = 0.8). Simulation dataset 4 introduces a covariance gradient in addition to simulation dataset 3. The gene correlation coefficient for genes with RANK >1.5 was set to R, the gene correlation coefficient for genes with 1.0 < RANK ≤1.5 was set to R/2, and the gene correlation coefficient for genes with RANK ≤1.0 was set to zero. Simulation dataset 4 was used to simulate situations in which genes with higher RANK had a higher degree of correlation, and genes with lower RANK had lower or no correlation (Figure 2D shows simulated data with R = 0.8).

The four weighting methods described in the Materials and Methods section were used to test the four sets of simulated data. For simulation data 1, the P-value and the covariance of the gene set and gene list showed a good response relationship, with a “U-shaped” line graph. As shown in Figure 4A, on both sides of covariance = 0, the P-value decreased rapidly with an increase in |covariance|, and no significant difference was observed among the four weighting methods. For simulation data 2, the overall shape of the line graph was different from that of simulation data 1; namely, it was “J-shaped.” Specifically, on the covariance (0, 1) side, the P-value decreased rapidly with an increase in |covariance|, which is consistent with simulation test data 1. By contrast, in the covariance (−1, 0) side, no marked decrease was observed in the P-value with an increase in |covariance|, and the results of the four weighting methods were highly consistent. For simulated data 3, the trend between the obtained P-value and the covariance of the gene set and gene list was similar to that of simulation data 1; namely, it was also “U-shaped,” that is, the P-value decreased rapidly with an increase in |covariance| on both sides of covariance = 0. The steepness of the curve was significantly lower than that of simulation data 1, and slight differences were observed between the four weighting methods. For simulated data 4, the curve between the P-value and the covariance of the gene set and gene list was also “U-shaped.” However, the steepness of the curve was flatter than that of simulated data 3, and the P-values obtained by the four weighting methods were significantly different. Specifically, the W7 method was the steepest, followed by the W1, W2, W8, and W0 methods.

### 3.4 Identification of cell line-specific key transcription factors

**FIGURE 3:**
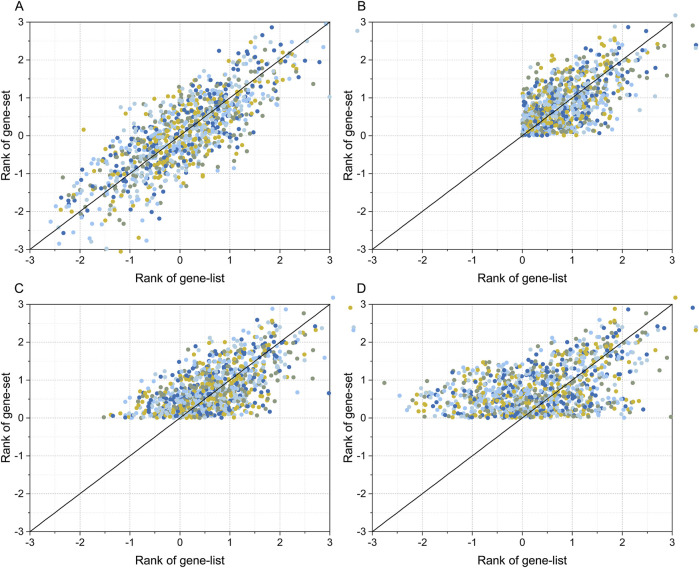
Simulated gene set and gene list datasets. The simulated data were generated using the “mvrnorm” function in R. Plot **(A)** shows the simulated dataset for a full-spectrum distribution with covariance (R) = 0.8. Plot **(B)** shows the simulated dataset with RANK >0 for both the gene list, gene set, and R = 0.8. Plot **(C)** shows the simulated dataset with RANK >0 for gene set, full-spectrum distribution for gene list, and R = 0.8. The dataset in plot **(D)** is the same as that of plot **(C)** except R is the gradient correlation (i.e. R = 0.8 for gene list >1.5, R = 0.4 for 1.0 < gene list ≤1.5, and R = 0 for gene list ≤1.0).

**FIGURE 4:**
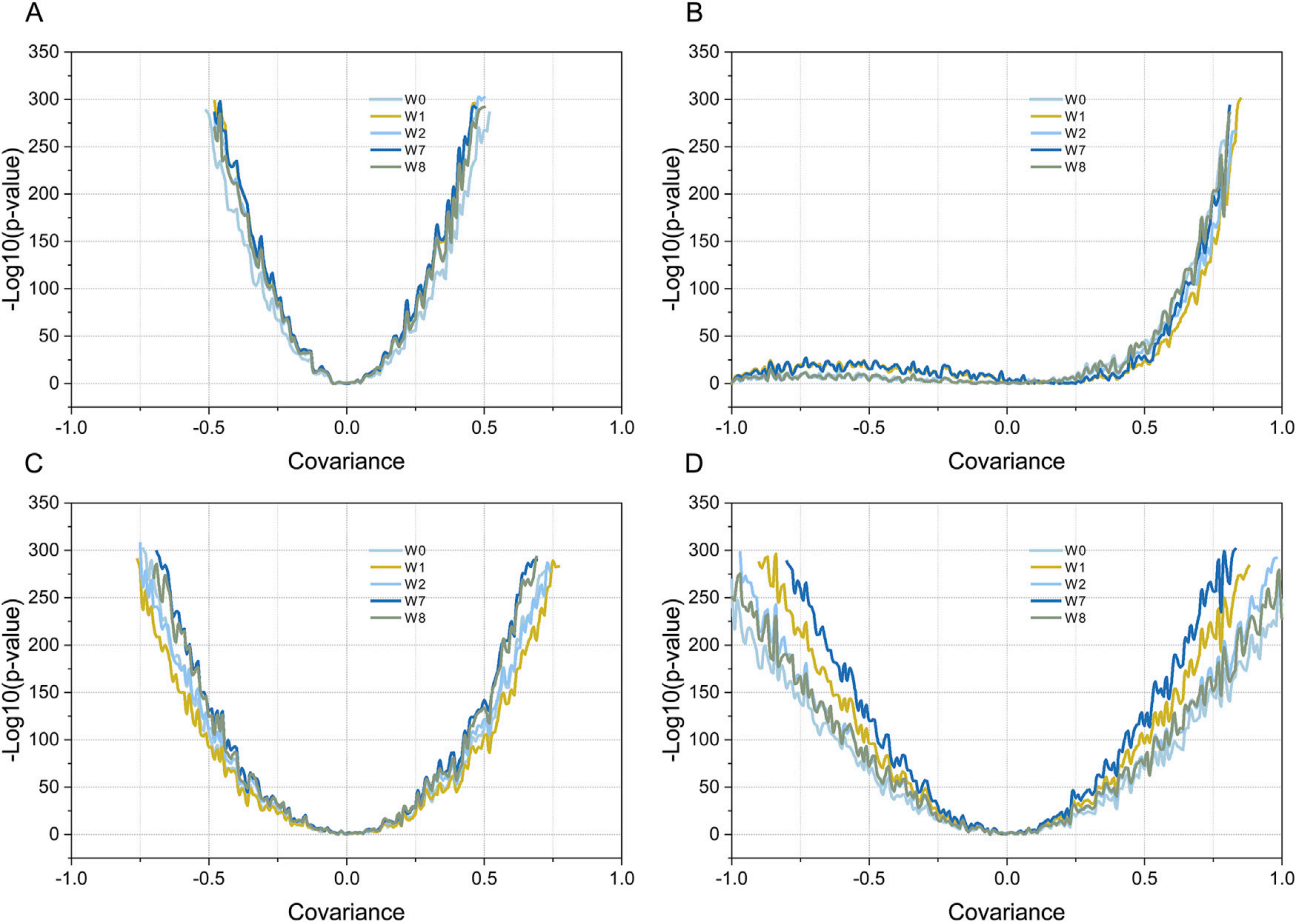
Testing simulated data using four different weighted Kendall’s tau correlation statistical methods. Plot **(A–D)** show the results of simulation data 1-4, respectively. Curves with different colors indicate the relationship between the P-values obtained by the different weighting methods and the covariance values (R). Dark blue, yellow, dark brown, light green, and light gray indicate the MIXED-DENSITY-CURVE (W7), DENSITY-CURVE (W1), MIX-LINEAR (W8), LINEAR (W2), and STD (W0) weighting methods, respectively.

**FIGURE 5:**
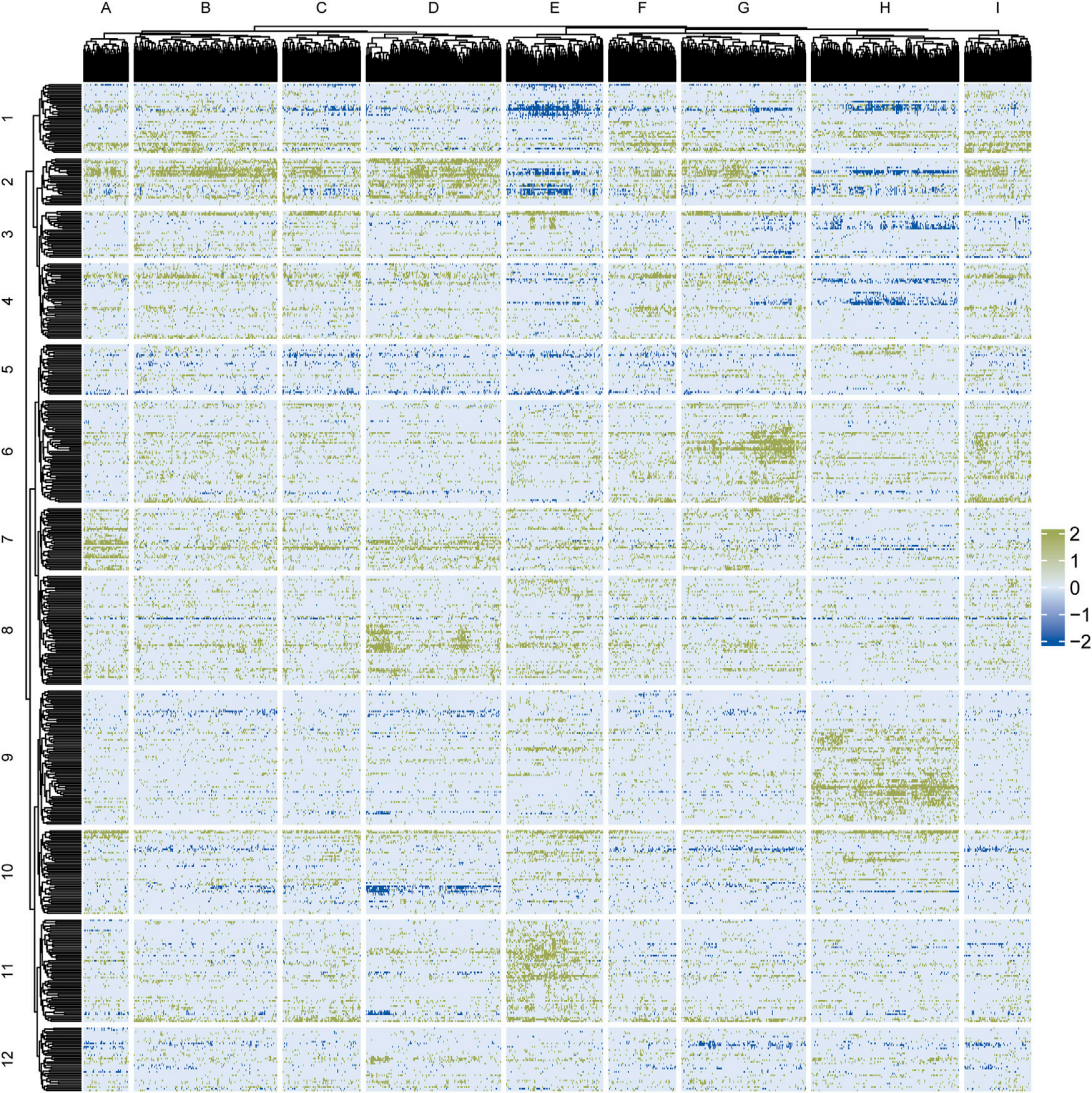
Clustering analysis of Flaver results for genome-wide transcriptome data of 1,206 cell lines. Cell line samples are shown in the columns, while transcription factors were shown in the rows. Green dots indicate positive correlations between gene set and gene list, while blue dots indicate negative correlations. Color depth was calculated by -Log10 (P-value) according to the Flaver’s statistics.

**FIGURE 6:**
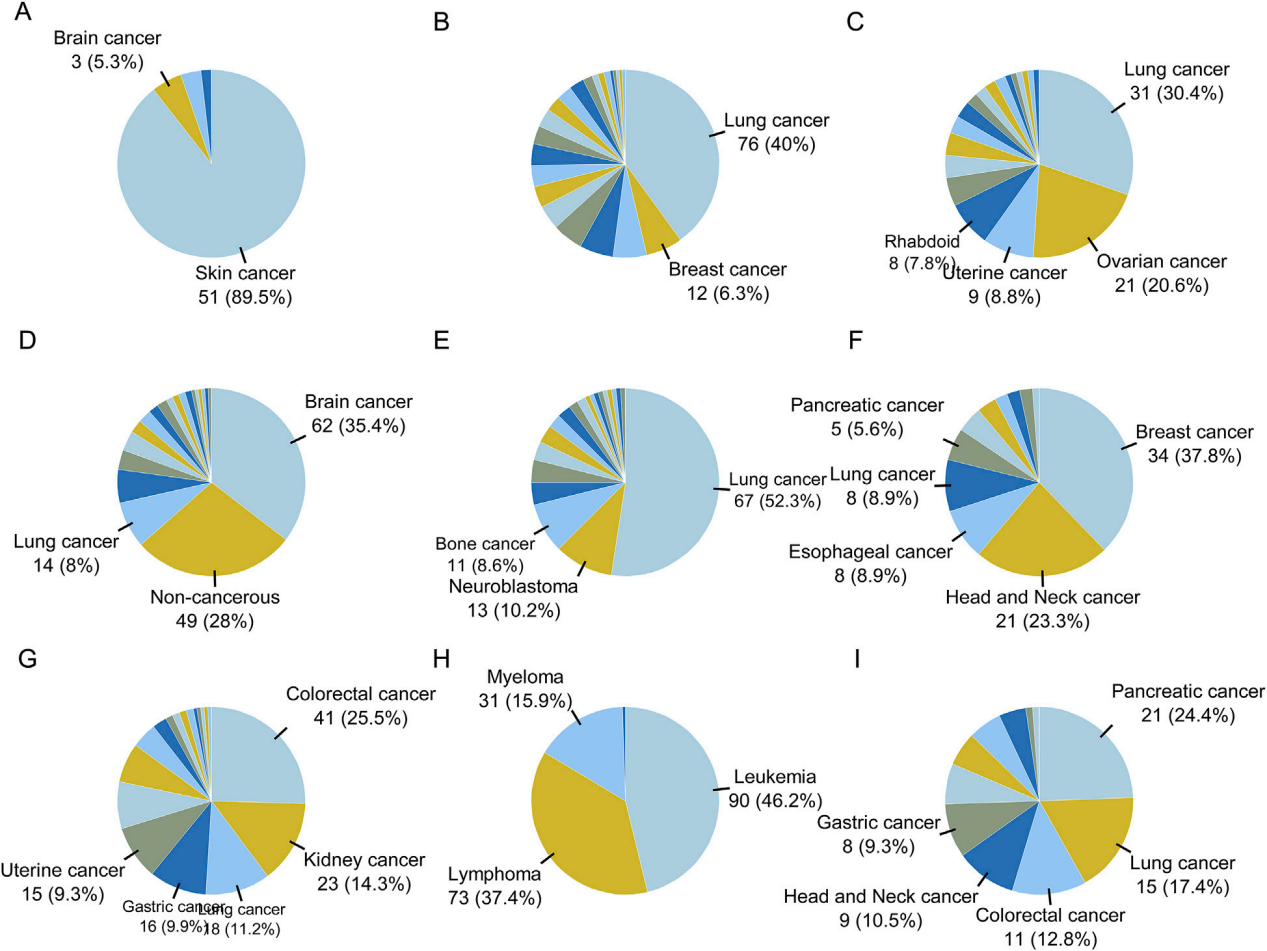
The proportion of disease types for cell lines in clusters obtained by Flaver analysis. The proportion of cell types for sample in each cluster are shown as pie graphs from **(A–I)**.

**FIGURE 7:**
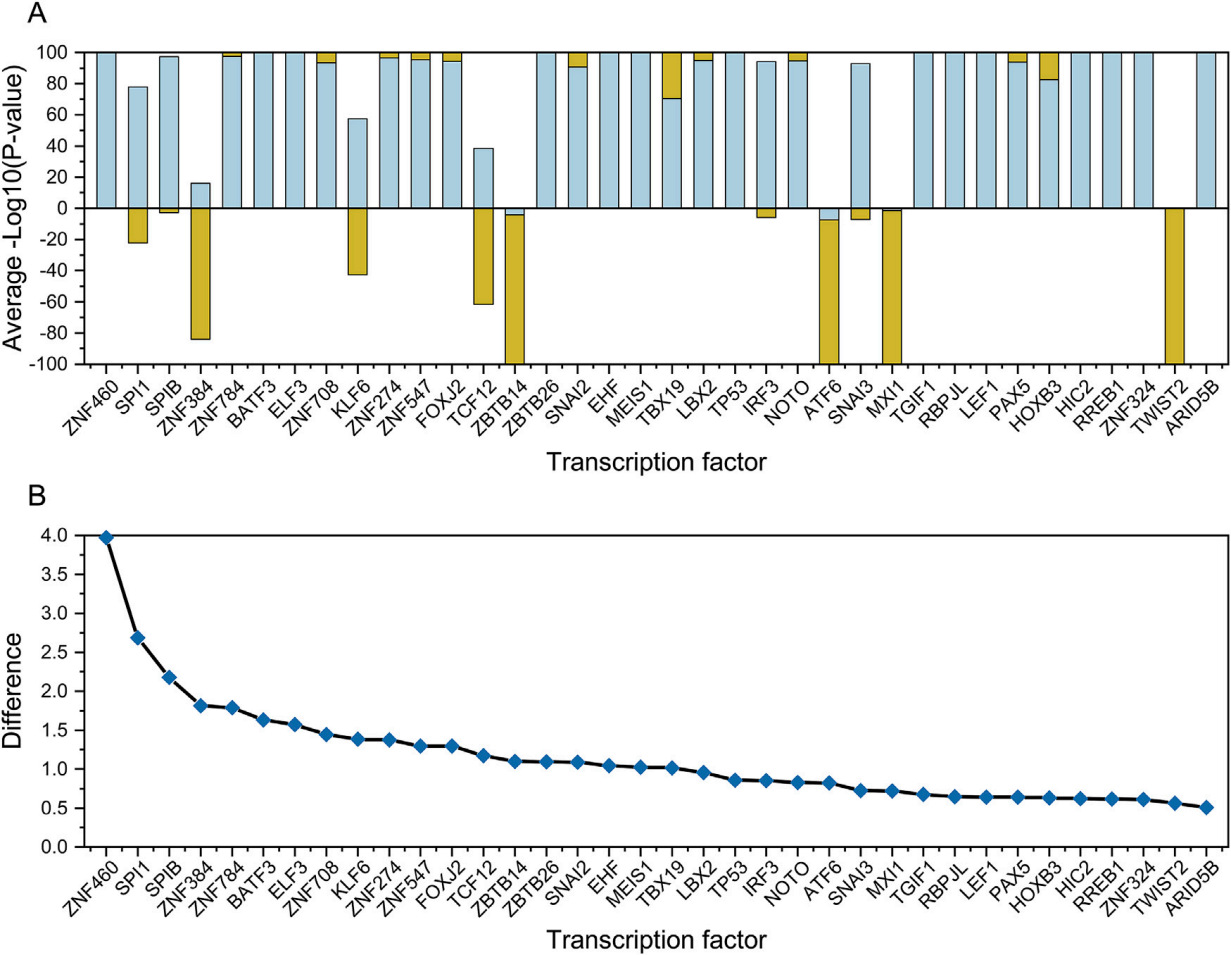
Comparative analysis of transcription factors between cancer and non-cancer groups. Plot **(A)** shows the normalized mean -Log10 (P-value) values for transcription factors between the cancer (orange) and non-cancer groups (light green). Plot **(B)** shows the difference between the cancer and non-cancer groups for each transcription factor, the top 6 of which were selected for wet-lab validation.

**FIGURE 8:**
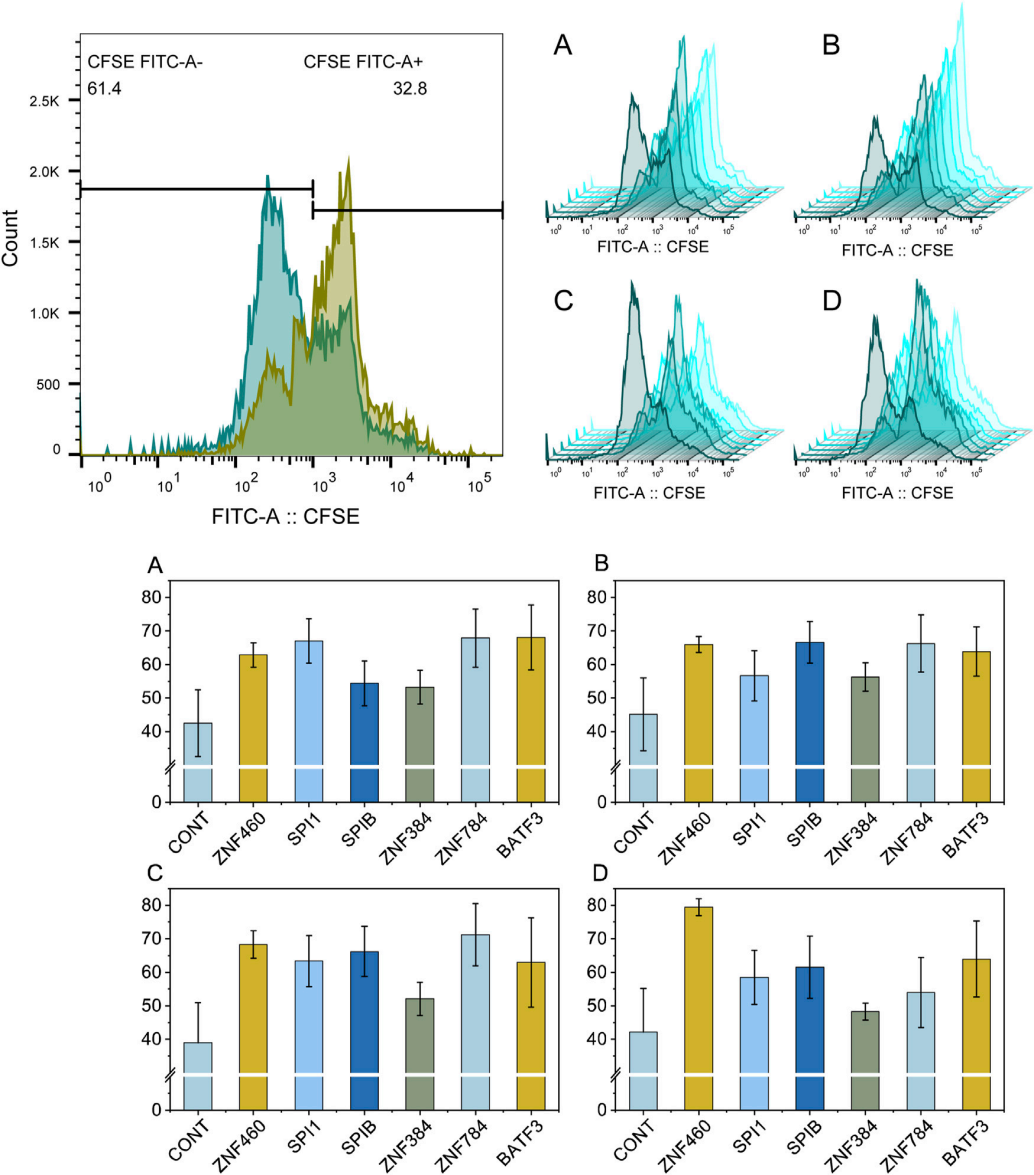
Flow cytometry analysis of the proliferation rate of leukemia cells treated with shRNA lentiviral vectors targeting key transcription factors. Plot **(A–D)** show the flow cytometry results for HL-60, MOLM-13, OCI-AML-3, U-937, respectively. The nontreated control samples and samples treated with transcription factors of ZNF460, SPI1, SPIB, ZNF384, ZNF784, and BATF3 are colored in dark green to light green, respectively. Each experiment was run in triplicate.

The Flaver software was used to identify key transcription factors in the sample-specific gene lists. The real-world gene list, CELLINE list, described in the Materials and Methods section, and the Jinzer set created in the Jinzer software, were used as input files for Flaver. The MIXED-DENSITY-CURVE function, which has been proven to be the most sensitive method (W7), was used to run the Flaver. A total of 644 transcription factors were identified within a running time of 23 h (FDR <0.05). Figure 5 shows the clustering results according to the sign (Dir) * -log (P-value) value of the transcription factors. Based on the Spearman’s rank correlation coefficient distance, the 1,206 cell lines were divided into nine categories (Figures 5A–I), and the transcription factors were divided into 12 categories (Figures 1–5, 5–12).

The proportions of cell line types in each category are plotted in Figure 6. Brain cancer samples were predominantly located in cluster D, while leukemia, lymphoma, and myeloma samples were mainly located in cluster H. Skin cancer samples were located in cluster A, breast cancer samples in class F, and colorectal cancer samples in clusters G and I. Head and neck cancer samples were mainly located in class F, while kidney cancer samples were distributed in cluster G. Uterine and ovarian cancer samples were distributed in cluster C. Lung cancer samples were found in clusters B, C, E, I, and D. Non-cancerous samples were mainly located in cluster D.

### 3.5 Regulator module discriminated cancerous and non-cancerous cell lines

From the clustering analysis results (Figure 5), we identified a set of transcription factors (row 9) that showed the same regulatory behavior in leukemia samples (column H). A significant positive correlation was observed between the RANK values of the gene set and the gene list for these transcription factors. This set of transcription factors could be used to distinguish leukemia cell lines from other cell types (Figure 7A). Based on the comparison of the differences in -Sign (P-value) * Log10(P-value) for these transcription factors between the leukemia cell lines and other types of cell lines, we selected six transcription factors with the highest differences between the leukemia and non-cancer groups for further experimental verification, namely ZNF460, SPI1, SPIB, ZNF384, ZNF784, and BATF3 (Figure 7B).

Four cell lines (HL-60, MOLM-13, OCI-AML-3, and U-937) were used for experimental validation. The results which were presented in Figure 8 showed that the ZNF460 shRNA lentiviral vector significantly inhibited the proliferation of HL-60, MOLM-13, OCI-AML-3, and U-937 cells, and that the ZNF460, SPI1, ZNF784, and BATF3 shRNA lentiviral vectors exerted strong inhibitory effects. The inhibition rates of ZNF460, SPIB, ZNF784, and BATF3 in MOLM-13 cells were over 20%. Only one transcription factor (ZNF384) lentiviral vector did not reach significance in the OCI-AML-3 cell lines. The ZNF460 lentivirus vector inhibited the proliferation of 80% of the U-937 cells.

## 4 Discussion

Accurately mining key transcription factors and elucidating their characteristics will help to determine the mechanism underlying the genetic regulation of the cell transcriptome (Meyer and Liu, 2020). This in turn requires the identification of the transcription factor binding sites and their activation patterns. By combining the activation and repression statuses of transcription factors and changes in the transcription levels of their target genes, we were able to identify the key transcription factors in the transcriptome (Yao et al., 2024). Under experimental conditions, high-throughput methods, such as RNA-seq, can be used to quantify transcription and non-transcription factors in the transcriptome simultaneously (Stark et al., 2019). Various well-developed transcription factor binding site prediction software and ChIP-Seq technologies have been used to identify a large number of candidate transcription factor binding sites (Maleki et al., 2020; MacQuarrie et al., 2011). This has in turn enabled the identification of key transcription factors by correlating their binding affinities to target genes with the expression levels of these genes. Recently, a comparison of four state-of-the-art key transcription factor mining tools found that the Flaver method, which is based on weighted Kendall’s tau correlation coefficients, showed an improved performance (Yao et al., 2024).

At present, there are three correlation analysis methods: Pearson’s correlation coefficient, Spearman’s correlation coefficient, and Kendall’s tau correlation coefficient. The prerequisite for applying the Pearson’s correlation coefficient is that the input numerical value is quantitative data with a normal distribution (Akoglu, 2018). The Spearman’s correlation coefficient can be used when the data do not follow a normal distribution (Pripp, 2018), while Kendall’s tau correlation coefficient can be used when the input data are ordered categorical variables (Arndt et al., 1999). In a typical transcriptome profiling experiment, we set a threshold for the degree of difference in gene expression levels to obtain a gene list (Huang et al., 2018; Huang et al., 2020a). In transcription factor binding site analysis experiments, the same strategy is often used, namely setting a penalty threshold for binding sites and selecting data higher than the threshold to identify candidate binding sites with a higher reliability (Huang et al., 2022; Grant et al., 2011). This situation can be accurately simulated using the data shown in Figure 3B. The test results of this study showed that an incomplete distribution profile of data similar to that shown in Figure 3B can cause fallacies when applying Kendall’s tau statistical analysis; that is, when both gene list and gene set are filtered with RANK > threshold (zero in Figure 3B), negatively correlated transcription factors cannot be detected. The reason for this fallacy is obvious: when the RANK threshold screening condition is set, the data in the RANK < threshold part is missing. Assuming that the RANK value of gene set to be analyzed is positive, and negatively correlated with the gene list, the distribution of the corresponding gene list values should be located in the RANK < threshold region. As mentioned above, if the RANK > threshold screening condition is set, this part is missing, making the negatively correlated genes undetectable. In fact, this can be rescued if either the gene set or the gene list has a full-spectrum distribution; the simulation data in Figures 3C, D illustrate the case when the simulated gene set has a semi-spectrum distribution and the gene list has a full-spectrum distribution. The results in Figures 3C, D are similar to those shown in Figure 3A, when both the gene set and gene list followed a full-spectrum normal distribution, and both positive and negative correlations could be detected without failure.

In this study, four methods were used to transform transcriptome data into a gene list. First, the star START method was used to successfully convert high-dimensional data into two-dimensional data. However, when applied to gene expression data, it did not resolve the issue of arranging the order of coordinate axes and estimating the degree of gene expression difference. As previously mentioned, the order of samples can be determined by the hierarchical clustering of gene expression data. The axes of each sample in the START system were arranged in the order determined by the cluster tree. Unique 2D scatter plots with self-organizing features can be constructed for specific transcriptome data. Then, the differential gene expression levels were measured by calculating the vertical distance between the data points and the coordinate axis in a two-dimensional scatter plot. The data points located near the axis with a long vertical distance from the origin were the dominantly expressed genes of the sample, whereas the data points located near the axis with a short vertical distance from the origin were the recessively expressed genes in the sample. However, the START method can only identify genes with RANK > zero in the gene list. Combined with the analysis results of the four simulation datasets shown in Figure 3, the gene list created by the START method suffered from the fallacy shown in Figure 4B during Flaver analysis. The gene list transformed using the SPM method also belongs to the incomplete skewed distribution data type, such that it will also suffer the “unable to detect negative correlation” fallacy. The ED and Hg methods are essentially the same, and the RANK values for Hg method are the log form of the ED method. According to the results presented in Figures 2A, B, the distribution of the gene list created using the ED and Hg method followed an approximately normal distribution. Therefore, theoretically, it conforms to the simulated data presented in Figures 3C, D, and thus does not commit the “unable to detect negative correlation” fallacy shown in Figure 3B.

A possible solution is presented by Flaver software, which emphasizing genes with a high RANK and de-emphasizing those with a low RANK by implementing weighted Kendall’s tau statistics to measure the correlation, an innovative feature that is well demonstrated in this paper. The data in Figure 3D simulated the gene set and gene list, which were correlated with gradient eco-efficiency; that is, the high RANK regions were highly correlated, the medium RANK regions were moderately correlated, and the low RANK regions were not correlated. The effects of the five weighting methods on this dataset were evident as they were significantly different from the uniformly correlated simulated data shown in Figure 3A. The STD method tested in this paper is a standard implementation of Kendall’s tau correlation coefficient, the MIX-LINEAR method is a mixed linear weighting formula based on RANK of both gene set and gene list, the LINEAR method is a linear weighting formula for gene list only, which have been discussed previously (Yao et al., 2024). The MIXED-DENSITY-CURVE method is a mixed density distribution curve weighting formula based on the RANK of both the gene set and the gene list, and the DENSITY-CURVE method is a density distribution curve weighting formula for the gene list only. From the evaluation results presented in Figure 4D, the MIXED-DENSITY-CURVE method was identified as the most sensitive, resulting in a steeper U-shaped curve than any of the other four weighting methods.

As a result of transcriptome profiling experiments, most genes in the genome were found to be expressed at average levels (approximately normal distribution) in cells; genes with specifically high and specifically low levels of expression only accounted for a small number of genes (Huang et al., 2020b; Conesa et al., 2016). Providing equal weights to all genes in a weighted Kendall’s tau correlation analysis may obscure the genes of greatest interest, namely those that are either highly and weakly expressed, resulting in inappropriate statistical inference (Yao et al., 2024). As shown in this study, the MIXED-DENSITY-CURVE method is a hybrid density distribution curve weighting formula based on the RANK of both the gene set and gene list, which is symmetrically distributed along the coordinate axis on both sides of the origin. Theoretically, the MIXED-DENSITY-CURVE method has advantages in emphasizing specifically highly expressed and specifically low expressed genes, as well as weakening non-differentially expressed genes. This is the essence of weighted Kendall’s tau rank correlation, namely its ability to avoid the artificial statistical biases caused by imbalanced weights for upregulated or downregulated genes.

## Data Availability

The original contributions presented in the study are included in the article/Supplementary Material, further inquiries can be directed to the corresponding authors.
