# Supplementary material for: Correlating gene expression levels with transcription factor binding sites facilitates identification of key transcription factors from transcriptome data
Source: Front Genet. 2024 Nov 29;15:1511456. doi: 10.3389/fgene.2024.1511456 (PMC11638204; doi:10.3389/fgene.2024.1511456)
Supplement: Supplementary file 1 [file Table1.docx]

Supplemental Document

1. **File information for supplemental data**

**URL:** [**http://www.thua45.cn/falver/supplemental_data2.zip**](http://www.thua45.cn/falver/supplemental_data2.zip)

Root/Jinzer_set.zip Gene set file

Root/Promoter_550bp.zip Promoter sequence

Root/Transcriptome_data.zip Transcriptome data for 1206 Human cell lines

Root/Software/Jinzer.exe Jinzer software

Root/Software/Flaver.exe Flaver software

Root/Software/Jinzer-1.0 Jinzer software source code

Root/Software/Flaver-1.0.3 Flaver software source code

Root/Simu_data/01.zip Simulated gene set and list files created by 01 method

Root/Simu_data/02.zip Simulated gene set and list files created by 02 method

Root/Simu_data/03.zip Simulated gene set and list files created by 03 method

Root/Simu_data/04.zip Simulated gene set and list files created by 04 method

Root/R_script/Create_gene_list.zip R script for creation of gene list

Root/R_script/Create_simu_data.R R script for creation of simulated data

Root/Celline_list/START.zip Gene list file created by DE method

Root/Celline_list/ED.zip Gene list file created by DE method

Root/Celline_list/SPM.zip Gene list file created by DE method

Root/Celline_list/PEM.zip Gene list file created by DE method

Root/Flaver_output/STD.zip Gene list file created by STD method

Root/Flaver_output/LINEAR.zip Gene list file created by LINEAR method

Root/Flaver_output/MIXED-LINEAR.zip Gene list file created by MIXED-LEANER method

Root/Flaver_output/DENSITY-CURVE.zip Gene list file created by DENSITY-CURVE method

Root/Flaver_output/MIXED-DENSITY-CURVE.zip Gene list file created by MIXED-DENSITY-CURVE method

1. **shRNA lentivirus used in this study (Merck)**

| Transcription factor | TCR clone ID | Target sequence | Vector |
| --- | --- | --- | --- |
| ZNF460 | TRCN0000014888 | GCCCTCATTCAACACTTCATT | pLKO.1 |
| SPI1 | TRCN0000020534 | CCTCCACATCCCGCTTCGCCT | pLKO.1 |
| SPIB | TRCN0000020554 | CCTCTGGGATTTCTTTGTCAT | pLKO.1 |
| ZNF384 | TRCN0000016533 | CCCGAGATGAATGACCCTTAT | pLKO.1 |
| ZNF784 | TRCN0000015618 | CACCTTCAACAACTCCTCCAA | pLKO.1 |
| BATF3 | TRCN0000017885 | GCACCTGACAGAGGCACTGAA | pLKO.1 |

1. **Supplemental Fig. 1**


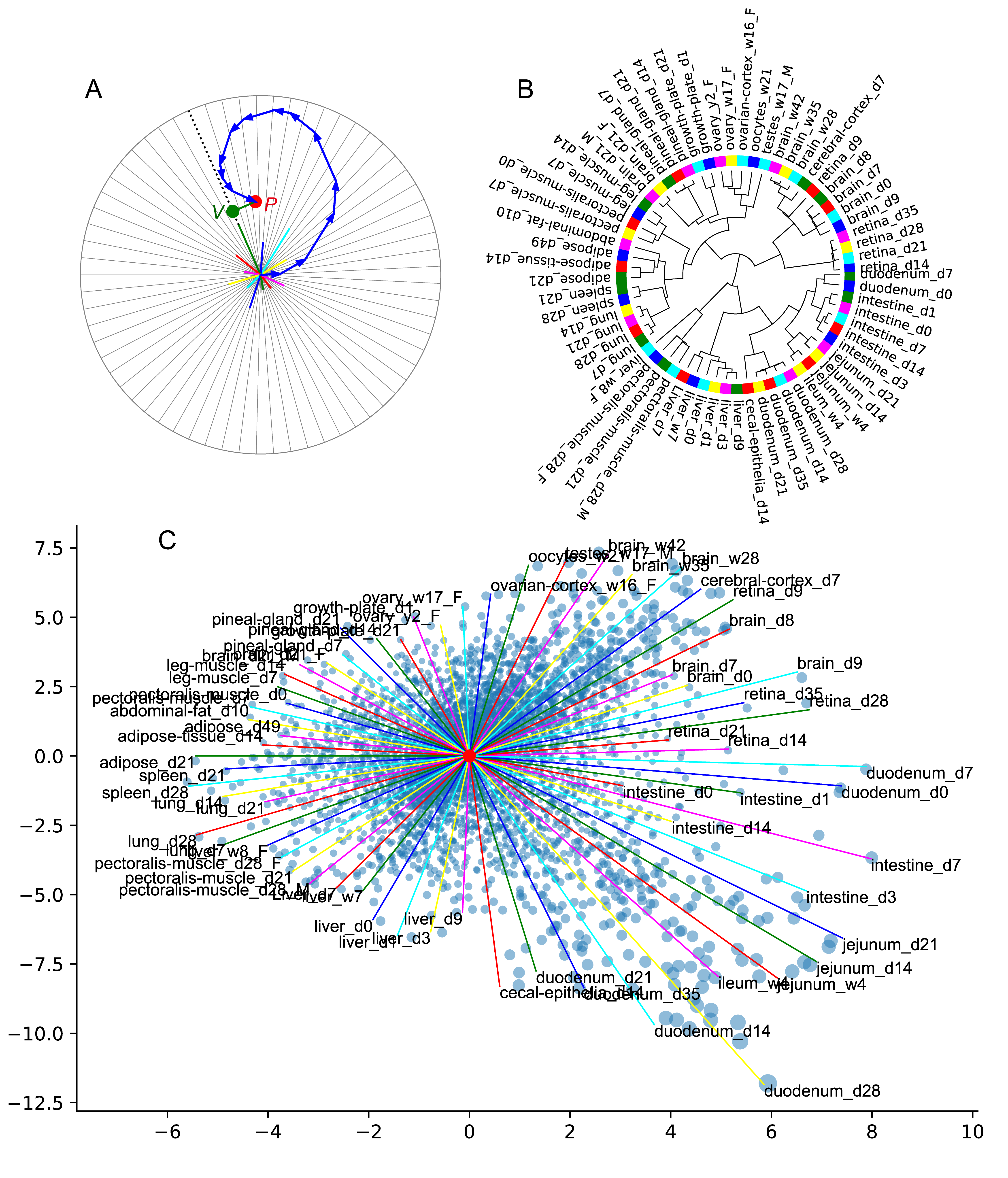


Fig. 1. Self-organized star-coordinate transformation method and an example for gene expression data. A schematic diagram of the calculation method for star-coordinate transformation, where the green arrow represents the result of adding the vectors specified by the expression values for each sample, starting from the origin site and ending at position P. V is the vertical intersection point from P to the coordinate axis of the target sample, and the length of VO is the measure of the degree of differential gene expression. B. The arrangement order of each coordinate axis determined by hierarchical clustering method. C. An example, the 2D scatter plot of the results of star-coordinate transformation of Human transcriptome data.

1. **Flow cytometry data**

**URL:** [**http://www.thua45.cn/falver/flow_cytometry_data.zip**](http://www.thua45.cn/falver/flow_cytometry_data.zip)

**Cells in vigorous division (5 × 106) were labeled with CFSE (Sigma-Aldrich) and then cocultured with shRNA lentivirus for 24 hours. Then the degree of cell division was gauged from the intensity of CFSE, as determined by flow cytometry (FITC channel). The ratio of CFSE+ cells to CFSE- cells was calculated.**

| **File name** | **Transcription factor** | **Cell line** | **Replication** | **File name** | **Transcription factor** | **Cell line** | **Replication** |
| --- | --- | --- | --- | --- | --- | --- | --- |
| 1001361 | Control | HL-60 | 1 | 1001421 | Control | ICI-AML-3 | 1 |
| 1001362 | Control | HL-60 | 2 | 1001422 | Control | ICI-AML-3 | 2 |
| 1001363 | Control | HL-60 | 3 | 1001423 | Control | ICI-AML-3 | 3 |
| 1001364 | ZNF460 | HL-60 | 1 | 1001424 | ZNF460 | ICI-AML-3 | 1 |
| 1001365 | ZNF460 | HL-60 | 2 | 1001425 | ZNF460 | ICI-AML-3 | 2 |
| 1001366 | ZNF460 | HL-60 | 3 | 1001426 | ZNF460 | ICI-AML-3 | 3 |
| 1001367 | SPI1 | HL-60 | 1 | 1001427 | SPI1 | ICI-AML-3 | 1 |
| 1001368 | SPI1 | HL-60 | 2 | 1001428 | SPI1 | ICI-AML-3 | 2 |
| 1001369 | SPI1 | HL-60 | 3 | 1001429 | SPI1 | ICI-AML-3 | 3 |
| 1001370 | SPIB | HL-60 | 1 | 1001430 | SPIB | ICI-AML-3 | 1 |
| 1001371 | SPIB | HL-60 | 2 | 1001431 | SPIB | ICI-AML-3 | 2 |
| 1001372 | SPIB | HL-60 | 3 | 1001432 | SPIB | ICI-AML-3 | 3 |
| 1001373 | ZNF384 | HL-60 | 1 | 1001433 | ZNF384 | ICI-AML-3 | 1 |
| 1001374 | ZNF384 | HL-60 | 2 | 1001434 | ZNF384 | ICI-AML-3 | 2 |
| 1001375 | ZNF384 | HL-60 | 3 | 1001435 | ZNF384 | ICI-AML-3 | 3 |
| 1001376 | ZNF784 | HL-60 | 1 | 1001436 | ZNF784 | ICI-AML-3 | 1 |
| 1001377 | ZNF784 | HL-60 | 2 | 1001437 | ZNF784 | ICI-AML-3 | 2 |
| 1001378 | ZNF784 | HL-60 | 3 | 1001438 | ZNF784 | ICI-AML-3 | 3 |
| 1001379 | BATF3 | HL-60 | 1 | 1001439 | BATF3 | ICI-AML-3 | 1 |
| 1001380 | BATF3 | HL-60 | 2 | 1001440 | BATF3 | ICI-AML-3 | 2 |
| 1001381 | BATF3 | HL-60 | 3 | 1001441 | BATF3 | ICI-AML-3 | 3 |
| 1001391 | Control | MOLM-13 | 1 | 1001421 | Control | U-937 | 1 |
| 1001392 | Control | MOLM-13 | 2 | 1001422 | Control | U-937 | 2 |
| 1001393 | Control | MOLM-13 | 3 | 1001423 | Control | U-937 | 3 |
| 1001394 | ZNF460 | MOLM-13 | 1 | 1001424 | ZNF460 | U-937 | 1 |
| 1001395 | ZNF460 | MOLM-13 | 2 | 1001425 | ZNF460 | U-937 | 2 |
| 1001396 | ZNF460 | MOLM-13 | 3 | 1001426 | ZNF460 | U-937 | 3 |
| 1001397 | SPI1 | MOLM-13 | 1 | 1001427 | SPI1 | U-937 | 1 |
| 1001398 | SPI1 | MOLM-13 | 2 | 1001428 | SPI1 | U-937 | 2 |
| 1001399 | SPI1 | MOLM-13 | 3 | 1001429 | SPI1 | U-937 | 3 |
| 1001400 | SPIB | MOLM-13 | 1 | 1001430 | SPIB | U-937 | 1 |
| 1001401 | SPIB | MOLM-13 | 2 | 1001431 | SPIB | U-937 | 2 |
| 1001402 | SPIB | MOLM-13 | 3 | 1001432 | SPIB | U-937 | 3 |
| 1001403 | ZNF384 | MOLM-13 | 1 | 1001433 | ZNF384 | U-937 | 1 |
| 1001404 | ZNF384 | MOLM-13 | 2 | 1001434 | ZNF384 | U-937 | 2 |
| 1001405 | ZNF384 | MOLM-13 | 3 | 1001435 | ZNF384 | U-937 | 3 |
| 1001406 | ZNF784 | MOLM-13 | 1 | 1001436 | ZNF784 | U-937 | 1 |
| 1001407 | ZNF784 | MOLM-13 | 2 | 1001437 | ZNF784 | U-937 | 2 |
| 1001408 | ZNF784 | MOLM-13 | 3 | 1001438 | ZNF784 | U-937 | 3 |
| 1001409 | BATF3 | MOLM-13 | 1 | 1001439 | BATF3 | U-937 | 1 |
| 1001410 | BATF3 | MOLM-13 | 2 | 1001440 | BATF3 | U-937 | 2 |
| 1001411 | BATF3 | MOLM-13 | 3 | 1001441 | BATF3 | U-937 | 3 |

1. **Columns (sample) information of clustering results**

| **Column** | **Cluster** | **Sample Index** | **Label** | **Descriprion** |
| --- | --- | --- | --- | --- |
| 1 | 1 | 282 | IGR.1 | Skin cancer |
| 2 | 1 | 885 | HT.144 | Skin cancer |
| 3 | 1 | 28 | Hs.294T | Skin cancer |
| 4 | 1 | 479 | A101D | Skin cancer |
| 5 | 1 | 496 | WM793 | Skin cancer |
| 6 | 1 | 300 | COLO.829 | Skin cancer |
| 7 | 1 | 664 | A2058 | Skin cancer |
| 8 | 1 | 96 | WM88 | Skin cancer |
| 9 | 1 | 144 | COLO.800 | Skin cancer |
| 10 | 1 | 454 | UCSD.242L | Skin cancer |
| 11 | 1 | 467 | WM983B | Skin cancer |
| 12 | 1 | 1156 | SH.4 | Skin cancer |
| 13 | 1 | 1038 | LN.464 | Brain cancer |
| 14 | 1 | 1103 | SK.MEL.28 | Skin cancer |
| 15 | 1 | 1178 | Mel.928 | Skin cancer |
| 16 | 1 | 465 | IGR.37 | Skin cancer |
| 17 | 1 | 1076 | X888.mel | #N/A |
| 18 | 1 | 57 | Hs.944.T | Skin cancer |
| 19 | 1 | 118 | RPMI.7951 | Skin cancer |
| 20 | 1 | 669 | MDA.MB.435 | Skin cancer |
| 21 | 1 | 1025 | MDA.MB.435S | Skin cancer |
| 22 | 1 | 564 | LN.229 | Brain cancer |
| 23 | 1 | 620 | A.375 | Skin cancer |
| 24 | 1 | 400 | UACC.62 | Skin cancer |
| 25 | 1 | 983 | SK.MEL.5 | Skin cancer |
| 26 | 1 | 348 | WM115 | Skin cancer |
| 27 | 1 | 602 | Hs.852.T | Skin cancer |
| 28 | 1 | 50 | HMY.1 | Skin cancer |
| 29 | 1 | 234 | K029AX | Skin cancer |
| 30 | 1 | 728 | COLO.699 | Lung cancer |
| 31 | 1 | 957 | SK.MEL.3 | Skin cancer |
| 32 | 1 | 92 | COLO.741 | Skin cancer |
| 33 | 1 | 556 | G.361 | Skin cancer |
| 34 | 1 | 881 | COLO.783 | Skin cancer |
| 35 | 1 | 1132 | DEOC.1 | Skin cancer |
| 36 | 1 | 283 | UACC.257 | Skin cancer |
| 37 | 1 | 1007 | Mel.JuSo | Skin cancer |
| 38 | 1 | 1095 | MeWo | Skin cancer |
| 39 | 1 | 247 | IPC.298 | Skin cancer |
| 40 | 1 | 394 | COLO.849 | Skin cancer |
| 41 | 1 | 337 | C32..Human.melanoma. | Skin cancer |
| 42 | 1 | 485 | SK.MEL.30 | Skin cancer |
| 43 | 1 | 638 | X537.mel | #N/A |
| 44 | 1 | 1041 | Hs.939.T | Skin cancer |
| 45 | 1 | 676 | COLO.792 | Skin cancer |
| 46 | 1 | 1139 | SK.MEL.23 | Skin cancer |
| 47 | 1 | 863 | Hs.936.T | Skin cancer |
| 48 | 1 | 1060 | X624.mel | #N/A |
| 49 | 1 | 840 | COLO.794 | Skin cancer |
| 50 | 1 | 1130 | Mel.Ho | Skin cancer |
| 51 | 1 | 397 | WM1799 | Skin cancer |
| 52 | 1 | 1011 | WM266.4 | Skin cancer |
| 53 | 1 | 411 | RVH.421 | Skin cancer |
| 54 | 1 | 713 | Malme.3M | Skin cancer |
| 55 | 1 | 103 | ONS.76 | Brain cancer |
| 56 | 1 | 980 | NCI.H2452 | Lung cancer |
| 57 | 1 | 1094 | HUVEC.TERT2 | Non-cancerous |
| 58 | 1 | 63 | SK.MEL.31 | Skin cancer |
| 59 | 1 | 99 | Hs.695T | Skin cancer |
| 60 | 1 | 402 | SK.MEL.24 | Skin cancer |
| 61 | 2 | 77 | JHOS.4 | Ovarian cancer |
| 62 | 2 | 156 | MDA.MB.468 | Breast cancer |
| 63 | 2 | 155 | COV413B | Ovarian cancer |
| 64 | 2 | 368 | OVSAHO | Ovarian cancer |
| 65 | 2 | 970 | COV362 | Ovarian cancer |
| 66 | 2 | 795 | COV504 | Ovarian cancer |
| 67 | 2 | 923 | HCC38 | Breast cancer |
| 68 | 2 | 986 | HCC1569 | Breast cancer |
| 69 | 2 | 370 | HCC1599 | Breast cancer |
| 70 | 2 | 517 | HCC2157 | Breast cancer |
| 71 | 2 | 1014 | NCI.H1819 | Lung cancer |
| 72 | 2 | 655 | MOR | Lung cancer |
| 73 | 2 | 820 | Caov.4 | Ovarian cancer |
| 74 | 2 | 971 | MOR.CPR | Lung cancer |
| 75 | 2 | 689 | RT.4 | Bladder cancer |
| 76 | 2 | 78 | HPAC | Pancreatic cancer |
| 77 | 2 | 832 | TE.5 | Esophageal cancer |
| 78 | 2 | 415 | NCI.H2228 | Lung cancer |
| 79 | 2 | 1028 | NCI.H1781 | Lung cancer |
| 80 | 2 | 897 | NCI.H1869 | Lung cancer |
| 81 | 2 | 272 | NCI.H3255 | Lung cancer |
| 82 | 2 | 497 | HCC2935 | Lung cancer |
| 83 | 2 | 1090 | KNS.62 | Lung cancer |
| 84 | 2 | 1173 | Detroit.562 | Head and Neck cancer |
| 85 | 2 | 597 | NCI.H596 | Lung cancer |
| 86 | 2 | 1123 | SNU.308 | Gallbladder cancer |
| 87 | 2 | 255 | NCI.H2110 | Lung cancer |
| 88 | 2 | 289 | NCI.H2122 | Lung cancer |
| 89 | 2 | 740 | NCI.H1437 | Lung cancer |
| 90 | 2 | 632 | NCI.H2170 | Lung cancer |
| 91 | 2 | 1198 | NCI.H3122 | Lung cancer |
| 92 | 2 | 1127 | HARA..Human.squa | Lung cancer |
| 93 | 2 | 583 | KYSE.140 | Esophageal cancer |
| 94 | 2 | 865 | KYSE.410 | Esophageal cancer |
| 95 | 2 | 765 | HCC2429 | Lung cancer |
| 96 | 2 | 242 | KYSE.180 | Esophageal cancer |
| 97 | 2 | 1179 | LC.1F | Lung cancer |
| 98 | 2 | 21 | NCI.H358 | Lung cancer |
| 99 | 2 | 176 | NCI.H2009 | Lung cancer |
| 100 | 2 | 271 | HSC.2 | Head and Neck cancer |
| 101 | 2 | 174 | OE21 | Esophageal cancer |
| 102 | 2 | 430 | TE.11 | Esophageal cancer |
| 103 | 2 | 139 | CAL.27 | Head and Neck cancer |
| 104 | 2 | 93 | SCaBER | Bladder cancer |
| 105 | 2 | 989 | HSC.3 | Head and Neck cancer |
| 106 | 2 | 339 | JHOS.2 | Ovarian cancer |
| 107 | 2 | 561 | HCC2270 | Lung cancer |
| 108 | 2 | 1100 | NCI.H2087 | Lung cancer |
| 109 | 2 | 925 | HCC827 | Lung cancer |
| 110 | 2 | 1193 | LCLC.97TM1 | Lung cancer |
| 111 | 2 | 544 | SW837 | Colorectal cancer |
| 112 | 2 | 877 | NCI.H747 | Colorectal cancer |
| 113 | 2 | 1122 | SW900 | Lung cancer |
| 114 | 2 | 912 | PK.45H | Pancreatic cancer |
| 115 | 2 | 1067 | RERF.LC.Ad1 | Lung cancer |
| 116 | 2 | 1206 | SNU.738 | Brain cancer |
| 117 | 2 | 305 | NCI.H1568 | Lung cancer |
| 118 | 2 | 666 | JIMT.1 | Breast cancer |
| 119 | 2 | 532 | MS751 | Cervical cancer |
| 120 | 2 | 706 | SW480 | Colorectal cancer |
| 121 | 2 | 76 | KPL.4 | Brain cancer |
| 122 | 2 | 133 | BT.20 | Breast cancer |
| 123 | 2 | 498 | SUM149PT | Breast cancer |
| 124 | 2 | 816 | SNU.216 | Gastric cancer |
| 125 | 2 | 244 | RL95.2 | Uterine cancer |
| 126 | 2 | 1119 | CAL.62 | Thyroid cancer |
| 127 | 2 | 869 | KP.2 | Pancreatic cancer |
| 128 | 2 | 879 | RT.112 | Bladder cancer |
| 129 | 2 | 314 | COLO.678 | Colorectal cancer |
| 130 | 2 | 802 | SNU.1196 | Bile duct cancer |
| 131 | 2 | 34 | KLM.1 | Pancreatic cancer |
| 132 | 2 | 493 | COLO.680N | Esophageal cancer |
| 133 | 2 | 178 | NUGC.2 | Gastric cancer |
| 134 | 2 | 1134 | CAL.29 | Bladder cancer |
| 135 | 2 | 1153 | NCI.H292 | Lung cancer |
| 136 | 2 | 613 | SNU.840 | Ovarian cancer |
| 137 | 2 | 66 | PC.14 | Lung cancer |
| 138 | 2 | 316 | PC.9 | Lung cancer |
| 139 | 2 | 519 | A.431 | Skin cancer |
| 140 | 2 | 766 | OE33 | Esophageal cancer |
| 141 | 2 | 536 | TE.15 | Esophageal cancer |
| 142 | 2 | 786 | SW780 | Bladder cancer |
| 143 | 2 | 385 | YD.10B | Head and Neck cancer |
| 144 | 2 | 920 | MKN74 | Gastric cancer |
| 145 | 2 | 390 | NCI.H322 | Lung cancer |
| 146 | 2 | 529 | HCC366 | Lung cancer |
| 147 | 2 | 675 | EBC.1 | Lung cancer |
| 148 | 2 | 683 | SW1271 | Lung cancer |
| 149 | 2 | 737 | MDA.MB.453 | Breast cancer |
| 150 | 2 | 924 | KYSE.150 | Esophageal cancer |
| 151 | 2 | 966 | KYSE.30 | Esophageal cancer |
| 152 | 2 | 160 | SLR20 | Bladder cancer |
| 153 | 2 | 831 | T24 | Bladder cancer |
| 154 | 2 | 586 | SNU.1 | Gastric cancer |
| 155 | 2 | 733 | RKO | Colorectal cancer |
| 156 | 2 | 408 | MSTO.211H | Lung cancer |
| 157 | 2 | 1033 | BT.12 | Rhabdoid |
| 158 | 2 | 192 | HCC44 | Lung cancer |
| 159 | 2 | 1051 | PSN1 | Pancreatic cancer |
| 160 | 2 | 936 | Lu.99 | Lung cancer |
| 161 | 2 | 198 | PaTu.8988t | Pancreatic cancer |
| 162 | 2 | 287 | SW1573 | Lung cancer |
| 163 | 2 | 909 | HCC2108 | Lung cancer |
| 164 | 2 | 788 | HCC1438 | Lung cancer |
| 165 | 2 | 1172 | NCI.H838 | Lung cancer |
| 166 | 2 | 377 | NCI.H650 | Lung cancer |
| 167 | 2 | 384 | NCI.H157 | Lung cancer |
| 168 | 2 | 1001 | HCC2450 | Lung cancer |
| 169 | 2 | 937 | NCI.H1793 | Lung cancer |
| 170 | 2 | 968 | NCI.H2882 | Lung cancer |
| 171 | 2 | 1163 | SH.10.TC | Gastric cancer |
| 172 | 2 | 31 | HCC2885 | Lung cancer |
| 173 | 2 | 521 | ACC.MESO.1 | Lung cancer |
| 174 | 2 | 201 | J82 | Bladder cancer |
| 175 | 2 | 464 | Calu.1 | Lung cancer |
| 176 | 2 | 19 | NCI.H2369 | Lung cancer |
| 177 | 2 | 38 | NCI.H2461 | Lung cancer |
| 178 | 2 | 829 | MPP.89 | Lung cancer |
| 179 | 2 | 566 | HCC95 | Lung cancer |
| 180 | 2 | 1054 | HOP.62 | Lung cancer |
| 181 | 2 | 302 | UM.UC.3 | Bladder cancer |
| 182 | 2 | 199 | HT.1080 | Sarcoma |
| 183 | 2 | 1204 | LOX.IMVI | Skin cancer |
| 184 | 2 | 26 | Ca.Ski | Cervical cancer |
| 185 | 2 | 1049 | PK.45P | Pancreatic cancer |
| 186 | 2 | 949 | TIME | Non-cancerous |
| 187 | 2 | 584 | PrEC.LH | Non-cancerous |
| 188 | 2 | 704 | NCI.H1915 | Lung cancer |
| 189 | 2 | 861 | MCF.10A | Non-cancerous |
| 190 | 2 | 165 | SALE | Non-cancerous |
| 191 | 2 | 1019 | HCC515 | Lung cancer |
| 192 | 2 | 367 | hTCEpi | Non-cancerous |
| 193 | 2 | 576 | HBEC3.KT | Non-cancerous |
| 194 | 2 | 636 | hTERT.HME1 | Non-cancerous |
| 195 | 2 | 806 | CJM..Human.mela | Skin cancer |
| 196 | 2 | 236 | SLR25 | Kidney cancer |
| 197 | 2 | 1189 | SNU.387 | Liver cancer |
| 198 | 2 | 853 | JHUEM.3 | Uterine cancer |
| 199 | 2 | 117 | OV56 | Ovarian cancer |
| 200 | 2 | 665 | Caki.1 | Kidney cancer |
| 201 | 2 | 696 | KMRC.1 | Kidney cancer |
| 202 | 2 | 560 | BICR.10 | Head and Neck cancer |
| 203 | 2 | 988 | SK.MES.1 | Lung cancer |
| 204 | 2 | 640 | NCI.H647 | Lung cancer |
| 205 | 2 | 1131 | NCI.H1792 | Lung cancer |
| 206 | 2 | 477 | TCCSUP | Bladder cancer |
| 207 | 2 | 902 | JJ012 | Bone cancer |
| 208 | 2 | 120 | DBTRG.05MG | Brain cancer |
| 209 | 2 | 264 | A.172 | Brain cancer |
| 210 | 2 | 1089 | HCC2279 | Lung cancer |
| 211 | 2 | 833 | IA.LM | Lung cancer |
| 212 | 2 | 1140 | NCI.H2135 | Lung cancer |
| 213 | 2 | 110 | MDA.MB.231 | Breast cancer |
| 214 | 2 | 119 | NCI.H1838 | Lung cancer |
| 215 | 2 | 712 | HCC1195 | Lung cancer |
| 216 | 2 | 369 | SK.RC.20 | Kidney cancer |
| 217 | 2 | 1066 | SLR24 | Kidney cancer |
| 218 | 2 | 616 | NCI.H1975 | Lung cancer |
| 219 | 2 | 815 | RERF.LC.Sq1 | Lung cancer |
| 220 | 2 | 487 | BC.3C | Bladder cancer |
| 221 | 2 | 914 | HCC1395 | Breast cancer |
| 222 | 2 | 124 | SK.HEP.1 | Liver cancer |
| 223 | 2 | 168 | NCI.H1355 | Lung cancer |
| 224 | 2 | 524 | NCI.H2023 | Lung cancer |
| 225 | 2 | 499 | HLF | Liver cancer |
| 226 | 2 | 1042 | COR.L105 | Lung cancer |
| 227 | 2 | 304 | LHCN.M2 | Non-cancerous |
| 228 | 2 | 746 | HHSteC | Uncategorized |
| 229 | 2 | 36 | EKVX | Lung cancer |
| 230 | 2 | 435 | NCI.H1563 | Lung cancer |
| 231 | 2 | 9 | OVCA420 | Ovarian cancer |
| 232 | 2 | 501 | JHH.4 | Liver cancer |
| 233 | 2 | 691 | SNU.886 | Liver cancer |
| 234 | 2 | 670 | BHT.101 | Thyroid cancer |
| 235 | 2 | 680 | OS.RC.2 | Kidney cancer |
| 236 | 2 | 673 | X8305C | #N/A |
| 237 | 2 | 814 | Hs.578T | Breast cancer |
| 238 | 2 | 994 | HOP.92 | Lung cancer |
| 239 | 2 | 16 | LMSU | Gastric cancer |
| 240 | 2 | 225 | BFTC.909 | Kidney cancer |
| 241 | 2 | 1004 | B.CPAP | Thyroid cancer |
| 242 | 2 | 73 | HCC461 | Lung cancer |
| 243 | 2 | 1024 | Hs.746.T | Gastric cancer |
| 244 | 2 | 631 | JHH.6 | Liver cancer |
| 245 | 2 | 1045 | NCI.H1755 | Lung cancer |
| 246 | 2 | 277 | HCC193 | Lung cancer |
| 247 | 2 | 1058 | GCT | Sarcoma |
| 248 | 2 | 1146 | RERF.LC.OK | Lung cancer |
| 249 | 2 | 1079 | FTC.133 | Thyroid cancer |
| 250 | 2 | 46 | FTC.238 | Thyroid cancer |
| 251 | 2 | 763 | LN.215 | Brain cancer |
| 252 | 3 | 320 | MDA.MB.436 | Breast cancer |
| 253 | 3 | 80 | H4 | Brain cancer |
| 254 | 3 | 643 | LCLC.103H | Lung cancer |
| 255 | 3 | 677 | Daoy | Brain cancer |
| 256 | 3 | 1074 | MDA.MB.157 | Breast cancer |
| 257 | 3 | 3 | RERF.LC.A1 | Lung cancer |
| 258 | 3 | 10 | NCI.H1623 | Lung cancer |
| 259 | 3 | 425 | NCI.H2172 | Lung cancer |
| 260 | 3 | 129 | NCI.H2596 | Lung cancer |
| 261 | 3 | 559 | IST.Mes1 | Lung cancer |
| 262 | 3 | 582 | NCI.H2804 | Lung cancer |
| 263 | 3 | 59 | NCI.H226 | Lung cancer |
| 264 | 3 | 109 | IST.Mes2 | Lung cancer |
| 265 | 3 | 571 | LXF.289 | Lung cancer |
| 266 | 3 | 216 | OVCAR.8 | Ovarian cancer |
| 267 | 3 | 543 | NCI.H1693 | Lung cancer |
| 268 | 3 | 752 | NCI.H2085 | Lung cancer |
| 269 | 3 | 1158 | NCI.H1650 | Lung cancer |
| 270 | 3 | 734 | TE.1 | Esophageal cancer |
| 271 | 3 | 739 | SK.OV.3 | Ovarian cancer |
| 272 | 3 | 358 | JHUEM.1 | Uterine cancer |
| 273 | 3 | 208 | PEO1 | Ovarian cancer |
| 274 | 3 | 522 | EFO.21 | Ovarian cancer |
| 275 | 3 | 274 | SNU.410 | Pancreatic cancer |
| 276 | 3 | 699 | NCI.H2405 | Lung cancer |
| 277 | 3 | 311 | MFE.319 | Uterine cancer |
| 278 | 3 | 947 | HEK293 | Non-cancerous |
| 279 | 3 | 233 | SBC.5 | Lung cancer |
| 280 | 3 | 326 | NCI.H1299 | Lung cancer |
| 281 | 3 | 1091 | SNU.668 | Gastric cancer |
| 282 | 3 | 404 | NCI.H23 | Lung cancer |
| 283 | 3 | 908 | KP.4 | Pancreatic cancer |
| 284 | 3 | 950 | NCI.H2286 | Lung cancer |
| 285 | 3 | 1203 | TE.617.T | Sarcoma |
| 286 | 3 | 961 | MES.SA | Sarcoma |
| 287 | 3 | 237 | EN | Uterine cancer |
| 288 | 3 | 1161 | NCI.H522 | Lung cancer |
| 289 | 3 | 181 | NCI.H1703 | Lung cancer |
| 290 | 3 | 197 | MFE.296 | Uterine cancer |
| 291 | 3 | 1061 | X639V | #N/A |
| 292 | 3 | 1138 | NCI.H2030 | Lung cancer |
| 293 | 3 | 15 | OVK18 | Ovarian cancer |
| 294 | 3 | 53 | SF172 | Brain cancer |
| 295 | 3 | 858 | MIA.PaCa.2 | Pancreatic cancer |
| 296 | 3 | 922 | ABC.1 | Lung cancer |
| 297 | 3 | 88 | OC.314 | Ovarian cancer |
| 298 | 3 | 574 | SUM1315MO2 | Breast cancer |
| 299 | 3 | 790 | LOU.NH91 | Lung cancer |
| 300 | 3 | 911 | HCC1576 | Adrenocortical cancer |
| 301 | 3 | 533 | C.33.A | Cervical cancer |
| 302 | 3 | 95 | HBL.100 | Uncategorized |
| 303 | 3 | 1126 | G.402 | Kidney cancer |
| 304 | 3 | 262 | MON | Rhabdoid |
| 305 | 3 | 762 | SK.UT.1 | Sarcoma |
| 306 | 3 | 375 | HEC.108 | Uterine cancer |
| 307 | 3 | 773 | HMC.1.8 | Breast cancer |
| 308 | 3 | 296 | KD..Human.abdomen | Rhabdoid |
| 309 | 3 | 753 | TTC.1240 | Rhabdoid |
| 310 | 3 | 826 | NCI.H2004RT | Rhabdoid |
| 311 | 3 | 1170 | KP.MRT.RY | Rhabdoid |
| 312 | 3 | 29 | COV434 | Ovarian cancer |
| 313 | 3 | 122 | TOV.112D | Ovarian cancer |
| 314 | 3 | 439 | SNU.398 | Liver cancer |
| 315 | 3 | 432 | HuTu.80 | Gastric cancer |
| 316 | 3 | 599 | SNU.484 | Gastric cancer |
| 317 | 3 | 447 | G.401 | Rhabdoid |
| 318 | 3 | 1073 | A2780 | Ovarian cancer |
| 319 | 3 | 720 | NCI.H1581 | Lung cancer |
| 320 | 3 | 779 | NCI.H2077 | Lung cancer |
| 321 | 3 | 659 | DMS.114 | Lung cancer |
| 322 | 3 | 716 | NCI.H841 | Lung cancer |
| 323 | 3 | 219 | A.427 | Lung cancer |
| 324 | 3 | 657 | PA.1 | Ovarian cancer |
| 325 | 3 | 240 | AN3.CA | Uterine cancer |
| 326 | 3 | 2 | A.204 | Rhabdoid |
| 327 | 3 | 112 | TTC.709 | Rhabdoid |
| 328 | 3 | 111 | MX.1 | Breast cancer |
| 329 | 3 | 153 | Kuramochi | Ovarian cancer |
| 330 | 3 | 747 | OVKATE | Ovarian cancer |
| 331 | 3 | 416 | OVCAR.3 | Ovarian cancer |
| 332 | 3 | 1157 | ONCO.DG.1 | Ovarian cancer |
| 333 | 3 | 462 | U2OS | Bone cancer |
| 334 | 3 | 203 | AF22 | Uncategorized |
| 335 | 3 | 1145 | SuSa | Testis cancer |
| 336 | 3 | 39 | OAW28 | Ovarian cancer |
| 337 | 3 | 85 | COV318 | Ovarian cancer |
| 338 | 3 | 644 | NCI.H1734 | Lung cancer |
| 339 | 3 | 1008 | KLE | Uterine cancer |
| 340 | 3 | 261 | MFE.280 | Uterine cancer |
| 341 | 3 | 688 | OVCA432 | Ovarian cancer |
| 342 | 3 | 717 | JHH.1 | Liver cancer |
| 343 | 3 | 438 | Caov.3 | Ovarian cancer |
| 344 | 3 | 674 | OVCAR.4 | Ovarian cancer |
| 345 | 3 | 239 | KYSE.270 | Esophageal cancer |
| 346 | 3 | 870 | HCC1534 | Lung cancer |
| 347 | 3 | 372 | HCC1171 | Lung cancer |
| 348 | 3 | 1147 | PANC.1 | Pancreatic cancer |
| 349 | 3 | 1197 | FU.OV.1 | Ovarian cancer |
| 350 | 3 | 509 | HOS | Bone cancer |
| 351 | 3 | 1152 | X143B | #N/A |
| 352 | 3 | 431 | HCC1162 | Uterine cancer |
| 353 | 3 | 503 | SNU.626 | Brain cancer |
| 354 | 3 | 646 | HAP1 | Leukemia |
| 355 | 3 | 1183 | EFO.27 | Ovarian cancer |
| 356 | 4 | 276 | HSkMC | Uncategorized |
| 357 | 4 | 417 | ASC2telo.differentiated | Uncategorized |
| 358 | 4 | 596 | ASC52telo | Non-cancerous |
| 359 | 4 | 468 | Hs.675.T | Non-cancerous |
| 360 | 4 | 1053 | Hs.172.T | Non-cancerous |
| 361 | 4 | 997 | DM.3 | Non-cancerous |
| 362 | 4 | 1200 | TE.125.T | Non-cancerous |
| 363 | 4 | 279 | T1.73 | Non-cancerous |
| 364 | 4 | 374 | TO.175.T | Non-cancerous |
| 365 | 4 | 211 | Hs.606.T | Non-cancerous |
| 366 | 4 | 494 | Hs.343.T | Non-cancerous |
| 367 | 4 | 288 | Hs.839.T | Non-cancerous |
| 368 | 4 | 907 | Hs.934.T | Non-cancerous |
| 369 | 4 | 238 | Hs.739.T | Non-cancerous |
| 370 | 4 | 702 | Hs.737.T | Non-cancerous |
| 371 | 4 | 445 | Hs.870.T | Non-cancerous |
| 372 | 4 | 486 | Hs.863.T | Non-cancerous |
| 373 | 4 | 736 | Hs.742.T | Non-cancerous |
| 374 | 4 | 622 | TE.159.T | Non-cancerous |
| 375 | 4 | 336 | Hs.819.T | Non-cancerous |
| 376 | 4 | 424 | Hs.822.T | Non-cancerous |
| 377 | 4 | 523 | Hs.600.T | Non-cancerous |
| 378 | 4 | 356 | Hs.688.A..T | Non-cancerous |
| 379 | 4 | 386 | RS.5 | Non-cancerous |
| 380 | 4 | 860 | Hs.255.T | Non-cancerous |
| 381 | 4 | 868 | Hs.751.T | Non-cancerous |
| 382 | 4 | 910 | Hs.274.T | Non-cancerous |
| 383 | 4 | 850 | Hs.698.T | Non-cancerous |
| 384 | 4 | 267 | Hs.616.T | Non-cancerous |
| 385 | 4 | 906 | Hs.834.T | Non-cancerous |
| 386 | 4 | 32 | Hs.821.T | Non-cancerous |
| 387 | 4 | 1097 | Hs.940.T | Non-cancerous |
| 388 | 4 | 114 | GOS.3 | Brain cancer |
| 389 | 4 | 285 | KNS.81 | Brain cancer |
| 390 | 4 | 5 | U.251MG | Brain cancer |
| 391 | 4 | 1081 | LN.235 | Brain cancer |
| 392 | 4 | 1026 | NCE.G.130 | Brain cancer |
| 393 | 4 | 812 | SNU.1105 | Brain cancer |
| 394 | 4 | 1017 | KALS.1 | Brain cancer |
| 395 | 4 | 338 | SNB.75 | Brain cancer |
| 396 | 4 | 945 | SNU.201 | Brain cancer |
| 397 | 4 | 1160 | CCF.STTG1 | Brain cancer |
| 398 | 4 | 771 | CAS.1 | Brain cancer |
| 399 | 4 | 107 | Hs.683 | Brain cancer |
| 400 | 4 | 1098 | SW1783 | Brain cancer |
| 401 | 4 | 510 | AM.38 | Brain cancer |
| 402 | 4 | 1141 | Rh18 | Sarcoma |
| 403 | 4 | 842 | Hs.706.T | Non-cancerous |
| 404 | 4 | 1077 | SW1353 | Bone cancer |
| 405 | 4 | 1205 | HuH.28 | Bile duct cancer |
| 406 | 4 | 511 | KNS.60 | Brain cancer |
| 407 | 4 | 761 | SNU.489 | Brain cancer |
| 408 | 4 | 190 | TM.31 | Brain cancer |
| 409 | 4 | 587 | LN.319 | Brain cancer |
| 410 | 4 | 405 | SJSA.1 | Bone cancer |
| 411 | 4 | 483 | NCI.H2722 | Lung cancer |
| 412 | 4 | 703 | NCI.H2373 | Lung cancer |
| 413 | 4 | 315 | G.292.clone.A141B1 | Bone cancer |
| 414 | 4 | 457 | TT2609.C02 | Thyroid cancer |
| 415 | 4 | 195 | JMSU.1 | Bladder cancer |
| 416 | 4 | 292 | IOMM.Lee | Brain cancer |
| 417 | 4 | 792 | SUM159PT | Breast cancer |
| 418 | 4 | 1106 | ES.2 | Ovarian cancer |
| 419 | 4 | 609 | TYK.nu | Ovarian cancer |
| 420 | 4 | 137 | NCI.H460 | Lung cancer |
| 421 | 4 | 882 | HD.MY.Z | Uncategorized |
| 422 | 4 | 1188 | RERF.LC.MS | Lung cancer |
| 423 | 4 | 539 | HCC1359 | Lung cancer |
| 424 | 4 | 1118 | GAMG | Brain cancer |
| 425 | 4 | 98 | JHOC.5 | Ovarian cancer |
| 426 | 4 | 226 | UOK101 | Kidney cancer |
| 427 | 4 | 847 | HEK.TE | Non-cancerous |
| 428 | 4 | 856 | UO.31 | Kidney cancer |
| 429 | 4 | 268 | NCE.G.84 | Brain cancer |
| 430 | 4 | 528 | PODO.SVTERT152 | Non-cancerous |
| 431 | 4 | 652 | PODO.TERT256 | Non-cancerous |
| 432 | 4 | 388 | S.117 | Sarcoma |
| 433 | 4 | 625 | X59M | #N/A |
| 434 | 4 | 1071 | NCI.H2595 | Lung cancer |
| 435 | 4 | 350 | JL.1 | Lung cancer |
| 436 | 4 | 690 | NCI.H2803 | Lung cancer |
| 437 | 4 | 678 | DOV13 | Ovarian cancer |
| 438 | 4 | 967 | NCI.H2810 | Lung cancer |
| 439 | 4 | 993 | IGR.39 | Skin cancer |
| 440 | 4 | 193 | KP.N.SI9s | Neuroblastoma |
| 441 | 4 | 412 | SK.N.SH | Neuroblastoma |
| 442 | 4 | 51 | CHP.212 | Neuroblastoma |
| 443 | 4 | 449 | SK.N.AS | Neuroblastoma |
| 444 | 4 | 136 | OVCA433 | Ovarian cancer |
| 445 | 4 | 1032 | MG.63 | Bone cancer |
| 446 | 4 | 108 | KS.1..Human.gliobl | Brain cancer |
| 447 | 4 | 200 | HMCB | Skin cancer |
| 448 | 4 | 917 | Calu.6 | Lung cancer |
| 449 | 4 | 668 | SNU.466 | Brain cancer |
| 450 | 4 | 841 | JHUEM.2 | Uterine cancer |
| 451 | 4 | 1065 | NCI.H2795 | Lung cancer |
| 452 | 4 | 1136 | ESS.1 | Uterine cancer |
| 453 | 4 | 433 | Hs.766T | Pancreatic cancer |
| 454 | 4 | 843 | X8505C | #N/A |
| 455 | 4 | 866 | CAL.120 | Breast cancer |
| 456 | 4 | 318 | NCE.G.121 | Brain cancer |
| 457 | 4 | 756 | YKG.1 | Brain cancer |
| 458 | 4 | 782 | BT.549 | Breast cancer |
| 459 | 4 | 43 | PC.3 | Prostate cancer |
| 460 | 4 | 252 | ML.1..Human.thyroid | Thyroid cancer |
| 461 | 4 | 964 | NCE.G.61 | Brain cancer |
| 462 | 4 | 1030 | NCE.G.59 | Brain cancer |
| 463 | 4 | 14 | GI.1 | Brain cancer |
| 464 | 4 | 656 | SF539 | Brain cancer |
| 465 | 4 | 463 | X42.MG.BA | #N/A |
| 466 | 4 | 1148 | LN.18 | Brain cancer |
| 467 | 4 | 828 | YD.8 | Head and Neck cancer |
| 468 | 4 | 962 | T98G | Brain cancer |
| 469 | 4 | 551 | SK.LMS.1 | Sarcoma |
| 470 | 4 | 918 | A.1207 | Brain cancer |
| 471 | 4 | 191 | NCE.G.44 | Brain cancer |
| 472 | 4 | 1023 | hTEC.SVTERT24.B | Non-cancerous |
| 473 | 4 | 1039 | NCE.G.140 | Brain cancer |
| 474 | 4 | 837 | SW579 | Thyroid cancer |
| 475 | 4 | 767 | U.87MG.ATCC | Brain cancer |
| 476 | 4 | 125 | SW982 | Sarcoma |
| 477 | 4 | 218 | SF126 | Brain cancer |
| 478 | 4 | 340 | Hs.888.T | Non-cancerous |
| 479 | 4 | 653 | Hs.38.T | Ovarian cancer |
| 480 | 4 | 72 | TIG.3.TD | Non-cancerous |
| 481 | 4 | 35 | Hs.229.T | Non-cancerous |
| 482 | 4 | 87 | Hs.840.T | Non-cancerous |
| 483 | 4 | 106 | Hs.618.T | Non-cancerous |
| 484 | 4 | 890 | Hs.895.T | Non-cancerous |
| 485 | 4 | 243 | Hs.729.T | Uncategorized |
| 486 | 4 | 700 | BJ1.hTERT | Non-cancerous |
| 487 | 4 | 319 | Hs.281.T | Non-cancerous |
| 488 | 4 | 738 | HLF.a | Non-cancerous |
| 489 | 4 | 422 | BJ..Human.fibroblast. | Non-cancerous |
| 490 | 4 | 755 | fHDF.TERT166 | Non-cancerous |
| 491 | 4 | 24 | BJ.hTERT..SV40.Large.T | #N/A |
| 492 | 4 | 131 | BJ.hTERT..SV40.Large.T | #N/A |
| 493 | 4 | 470 | HBF.TERT88 | Non-cancerous |
| 494 | 4 | 1195 | hTERT.RPE1 | Non-cancerous |
| 495 | 4 | 353 | NHAHTDD | Non-cancerous |
| 496 | 4 | 389 | CH.157MN | Brain cancer |
| 497 | 4 | 64 | SK.LU.1 | Lung cancer |
| 498 | 4 | 1022 | LN.340 | Brain cancer |
| 499 | 4 | 1029 | U.343MGa | Brain cancer |
| 500 | 4 | 835 | HEY | Ovarian cancer |
| 501 | 4 | 894 | HEY.A8 | Ovarian cancer |
| 502 | 4 | 518 | RKN | Sarcoma |
| 503 | 4 | 725 | MDST8 | Colorectal cancer |
| 504 | 4 | 406 | NCE.G.118 | Brain cancer |
| 505 | 4 | 345 | NMC.G1 | Brain cancer |
| 506 | 4 | 346 | NCI.H2052 | Lung cancer |
| 507 | 4 | 399 | NCE.G.111 | Brain cancer |
| 508 | 4 | 1035 | SF295 | Brain cancer |
| 509 | 4 | 42 | U.178MG | Brain cancer |
| 510 | 4 | 663 | U.138MG | Brain cancer |
| 511 | 4 | 783 | U.118MG | Brain cancer |
| 512 | 4 | 173 | NCE.G.124 | Brain cancer |
| 513 | 4 | 867 | NCE.G.122 | Brain cancer |
| 514 | 4 | 900 | GMS.10 | Brain cancer |
| 515 | 4 | 992 | OV7 | Ovarian cancer |
| 516 | 4 | 976 | NCE.G.22 | Brain cancer |
| 517 | 4 | 520 | NCE.G.28T | Brain cancer |
| 518 | 4 | 1114 | NCE.G.120 | Brain cancer |
| 519 | 4 | 915 | SW1088 | Brain cancer |
| 520 | 4 | 27 | KG.1.C | Brain cancer |
| 521 | 4 | 955 | GB.1 | Brain cancer |
| 522 | 4 | 570 | M059K | Brain cancer |
| 523 | 4 | 768 | LN.382 | Brain cancer |
| 524 | 4 | 781 | LN.428 | Brain cancer |
| 525 | 4 | 873 | SaOS.2 | Bone cancer |
| 526 | 4 | 166 | SNU.423 | Liver cancer |
| 527 | 4 | 291 | SF268 | Brain cancer |
| 528 | 4 | 572 | NCI.H196 | Lung cancer |
| 529 | 4 | 206 | SNU.475 | Liver cancer |
| 530 | 4 | 801 | JHOM.1 | Ovarian cancer |
| 531 | 4 | 1190 | CAL.78 | Bone cancer |
| 532 | 4 | 650 | YH.13 | Brain cancer |
| 533 | 4 | 1162 | DK.MG | Brain cancer |
| 534 | 4 | 554 | LN.443 | Brain cancer |
| 535 | 4 | 905 | U.2197 | Sarcoma |
| 536 | 5 | 60 | SK.PN.DW | Bone cancer |
| 537 | 5 | 546 | NCI.H211 | Lung cancer |
| 538 | 5 | 1155 | QGP.1 | Pancreatic cancer |
| 539 | 5 | 567 | NCI.H889 | Lung cancer |
| 540 | 5 | 540 | COR.L47 | Lung cancer |
| 541 | 5 | 954 | NCI.H146 | Lung cancer |
| 542 | 5 | 132 | DMS.454 | Lung cancer |
| 543 | 5 | 731 | BEN | Lung cancer |
| 544 | 5 | 194 | VMRC.LCD | Lung cancer |
| 545 | 5 | 420 | UMC.11 | Lung cancer |
| 546 | 5 | 594 | NCI.H810 | Lung cancer |
| 547 | 5 | 862 | NCI.H2066 | Lung cancer |
| 548 | 5 | 892 | NCI.H716 | Colorectal cancer |
| 549 | 5 | 874 | RPMI.2650 | Head and Neck cancer |
| 550 | 5 | 4 | NCI.H727 | Lung cancer |
| 551 | 5 | 934 | HCC1833 | Lung cancer |
| 552 | 5 | 623 | TT | Thyroid cancer |
| 553 | 5 | 635 | NCI.H1385 | Lung cancer |
| 554 | 5 | 82 | NCI.H1876 | Lung cancer |
| 555 | 5 | 1052 | NCI.H1882 | Lung cancer |
| 556 | 5 | 1082 | DMS.153 | Lung cancer |
| 557 | 5 | 1085 | COR.L95 | Lung cancer |
| 558 | 5 | 502 | DMS.53 | Lung cancer |
| 559 | 5 | 461 | NCI.H2029 | Lung cancer |
| 560 | 5 | 557 | COLO.668 | Lung cancer |
| 561 | 5 | 745 | NCI.H209 | Lung cancer |
| 562 | 5 | 489 | NCI.H1436 | Lung cancer |
| 563 | 5 | 555 | NCI.H1618 | Lung cancer |
| 564 | 5 | 1018 | NCI.H1836 | Lung cancer |
| 565 | 5 | 548 | HCC33 | Lung cancer |
| 566 | 5 | 30 | NCI.H1105 | Lung cancer |
| 567 | 5 | 210 | NCI.H1184 | Lung cancer |
| 568 | 5 | 256 | DMS.273 | Lung cancer |
| 569 | 5 | 231 | NCI.H2106 | Lung cancer |
| 570 | 5 | 381 | NCI.H1770 | Lung cancer |
| 571 | 5 | 1137 | NCI.H847 | Lung cancer |
| 572 | 5 | 612 | COR.L24 | Lung cancer |
| 573 | 5 | 996 | CPC.N | Lung cancer |
| 574 | 5 | 246 | CHP.126 | Neuroblastoma |
| 575 | 5 | 436 | KP.N.RT.BM.1 | Neuroblastoma |
| 576 | 5 | 183 | NH.6 | Neuroblastoma |
| 577 | 5 | 772 | KP.N.YN | Neuroblastoma |
| 578 | 5 | 407 | SK.N.BE.2. | Neuroblastoma |
| 579 | 5 | 727 | MHH.NB.11 | Neuroblastoma |
| 580 | 5 | 91 | SH.SY5Y | Neuroblastoma |
| 581 | 5 | 333 | SiMa | Neuroblastoma |
| 582 | 5 | 624 | SK.N.FI | Neuroblastoma |
| 583 | 5 | 18 | SK.N.DZ | Neuroblastoma |
| 584 | 5 | 298 | IMR.32 | Neuroblastoma |
| 585 | 5 | 784 | Kelly | Neuroblastoma |
| 586 | 5 | 592 | NCI.H2227 | Lung cancer |
| 587 | 5 | 770 | NCI.H187 | Lung cancer |
| 588 | 5 | 22 | NCI.H345 | Lung cancer |
| 589 | 5 | 959 | NCI.H2081 | Lung cancer |
| 590 | 5 | 473 | NCI.H1930 | Lung cancer |
| 591 | 5 | 452 | COR.L279 | Lung cancer |
| 592 | 5 | 888 | NCI.H69 | Lung cancer |
| 593 | 5 | 253 | NCI.H510A | Lung cancer |
| 594 | 5 | 335 | SCLC.21H | Lung cancer |
| 595 | 5 | 360 | SCLC.22H | Lung cancer |
| 596 | 5 | 128 | NCI.H1155 | Lung cancer |
| 597 | 5 | 848 | DMS.79 | Lung cancer |
| 598 | 5 | 254 | NCI.H524 | Lung cancer |
| 599 | 5 | 569 | NCI.H1694 | Lung cancer |
| 600 | 5 | 824 | NCI.H2171 | Lung cancer |
| 601 | 5 | 6 | NCI.N417 | Lung cancer |
| 602 | 5 | 939 | NCI.H446 | Lung cancer |
| 603 | 5 | 149 | ECC12 | Gastric cancer |
| 604 | 5 | 1036 | D341.Med | Brain cancer |
| 605 | 5 | 130 | D283.Med | Brain cancer |
| 606 | 5 | 478 | NCI.H82 | Lung cancer |
| 607 | 5 | 266 | COLO.684 | Uterine cancer |
| 608 | 5 | 611 | COLO.704 | Ovarian cancer |
| 609 | 5 | 270 | NCI.H526 | Lung cancer |
| 610 | 5 | 990 | COR.L311 | Lung cancer |
| 611 | 5 | 1063 | CADO.ES1 | Bone cancer |
| 612 | 5 | 48 | NCI.H1092 | Lung cancer |
| 613 | 5 | 775 | NCI.H1963 | Lung cancer |
| 614 | 5 | 601 | NCI.H660 | Prostate cancer |
| 615 | 5 | 697 | COR.L88 | Lung cancer |
| 616 | 5 | 61 | NCI.H2198 | Lung cancer |
| 617 | 5 | 169 | NCI.H2196 | Lung cancer |
| 618 | 5 | 371 | NCI.H661 | Lung cancer |
| 619 | 5 | 471 | KYM.1 | Rhabdoid |
| 620 | 5 | 148 | NCI.H520 | Lung cancer |
| 621 | 5 | 581 | LK.2 | Lung cancer |
| 622 | 5 | 754 | KNS.42 | Brain cancer |
| 623 | 5 | 1185 | NCI.H1048 | Lung cancer |
| 624 | 5 | 351 | RD.ES | Bone cancer |
| 625 | 5 | 719 | SK.NEP.1 | Bone cancer |
| 626 | 5 | 904 | SK.N.MC | Bone cancer |
| 627 | 5 | 307 | TC.71 | Bone cancer |
| 628 | 5 | 1192 | A.673 | Bone cancer |
| 629 | 5 | 1112 | EW.8 | Bone cancer |
| 630 | 5 | 361 | MHH.ES.1 | Bone cancer |
| 631 | 5 | 344 | SK.ES.1 | Bone cancer |
| 632 | 5 | 864 | EWS.502 | Bone cancer |
| 633 | 5 | 729 | TTC.549 | Rhabdoid |
| 634 | 5 | 844 | RD | Sarcoma |
| 635 | 5 | 1113 | LXFL.529L | Lung cancer |
| 636 | 5 | 999 | NCI.H1341 | Lung cancer |
| 637 | 5 | 104 | Rh41 | Sarcoma |
| 638 | 5 | 817 | Rh30 | Sarcoma |
| 639 | 5 | 7 | STM91.01 | Rhabdoid |
| 640 | 5 | 1027 | NTERA.2 | Uncategorized |
| 641 | 5 | 75 | HGC.27 | Gastric cancer |
| 642 | 5 | 859 | NB1 | Neuroblastoma |
| 643 | 5 | 705 | SNU.182 | Liver cancer |
| 644 | 5 | 827 | Tm87.16 | Rhabdoid |
| 645 | 5 | 429 | X22Rv1 | #N/A |
| 646 | 5 | 363 | MDA.PCa.2b | Prostate cancer |
| 647 | 5 | 871 | VCaP | Prostate cancer |
| 648 | 5 | 434 | LNCaP.clone.FGC | Prostate cancer |
| 649 | 5 | 825 | LNCaP | Prostate cancer |
| 650 | 5 | 972 | GSS | Gastric cancer |
| 651 | 5 | 1133 | SK.MEL.1 | Skin cancer |
| 652 | 5 | 480 | COLO.320 | Colorectal cancer |
| 653 | 5 | 948 | COLO.320DM | Colorectal cancer |
| 654 | 5 | 760 | SHP.77 | Lung cancer |
| 655 | 5 | 392 | DU4475 | Breast cancer |
| 656 | 5 | 724 | ECC10 | Gastric cancer |
| 657 | 5 | 84 | TTC.642 | Rhabdoid |
| 658 | 5 | 357 | TE.441.T | Sarcoma |
| 659 | 5 | 1120 | DV.90 | Lung cancer |
| 660 | 5 | 1121 | NCI.H1395 | Lung cancer |
| 661 | 5 | 263 | SK.MEL.2 | Skin cancer |
| 662 | 5 | 805 | RPMI.8226 | Myeloma |
| 663 | 5 | 365 | HCC1187 | Breast cancer |
| 664 | 5 | 834 | SU.DHL.1 | Lymphoma |
| 665 | 6 | 891 | HCC1482 | Cervical cancer |
| 666 | 6 | 893 | MDA.MB.134.VI | Breast cancer |
| 667 | 6 | 451 | HCC1428 | Breast cancer |
| 668 | 6 | 774 | MDA.MB.361 | Breast cancer |
| 669 | 6 | 651 | MDA.MB.330 | Breast cancer |
| 670 | 6 | 780 | HCC1419 | Breast cancer |
| 671 | 6 | 935 | MDA.MB.175.VII | Breast cancer |
| 672 | 6 | 460 | MDA.MB.415 | Breast cancer |
| 673 | 6 | 1021 | HCC2688 | Breast cancer |
| 674 | 6 | 418 | SK.BR.3 | Breast cancer |
| 675 | 6 | 1010 | AU565 | Breast cancer |
| 676 | 6 | 25 | HCC202 | Breast cancer |
| 677 | 6 | 507 | T.47d | Breast cancer |
| 678 | 6 | 614 | TE.4 | Esophageal cancer |
| 679 | 6 | 1055 | MFM.223 | Breast cancer |
| 680 | 6 | 672 | CAMA.1 | Breast cancer |
| 681 | 6 | 17 | ZR.75.30 | Breast cancer |
| 682 | 6 | 1096 | HCC2911 | Breast cancer |
| 683 | 6 | 444 | ZR.75.1 | Breast cancer |
| 684 | 6 | 715 | YMB.1 | Breast cancer |
| 685 | 6 | 506 | UACC.893 | Breast cancer |
| 686 | 6 | 932 | EFM.192A | Breast cancer |
| 687 | 6 | 134 | HCC1500 | Breast cancer |
| 688 | 6 | 257 | BT.483 | Breast cancer |
| 689 | 6 | 759 | EFM.19 | Breast cancer |
| 690 | 6 | 11 | Evsa.T | Breast cancer |
| 691 | 6 | 946 | BT.474 | Breast cancer |
| 692 | 6 | 322 | MCF.7 | Breast cancer |
| 693 | 6 | 822 | KPL.1 | Breast cancer |
| 694 | 6 | 205 | HCC2218 | Breast cancer |
| 695 | 6 | 1174 | CAL.148 | Breast cancer |
| 696 | 6 | 359 | Karpas.299 | Lymphoma |
| 697 | 6 | 482 | SCC.3 | Lymphoma |
| 698 | 6 | 281 | HCC4017 | Lung cancer |
| 699 | 6 | 1047 | HMEL | Non-cancerous |
| 700 | 6 | 813 | SNU.899 | Head and Neck cancer |
| 701 | 6 | 177 | CAL.33 | Head and Neck cancer |
| 702 | 6 | 630 | BICR.22 | Head and Neck cancer |
| 703 | 6 | 491 | ChaGo.K.1 | Lung cancer |
| 704 | 6 | 1093 | KYSE.70 | Esophageal cancer |
| 705 | 6 | 617 | KYSE.450 | Esophageal cancer |
| 706 | 6 | 1194 | FaDu | Head and Neck cancer |
| 707 | 6 | 329 | TE.9 | Esophageal cancer |
| 708 | 6 | 469 | SNU.503 | Colorectal cancer |
| 709 | 6 | 1110 | T.T | Esophageal cancer |
| 710 | 6 | 241 | LUDLU.1 | Lung cancer |
| 711 | 6 | 764 | EC.GI.10 | Esophageal cancer |
| 712 | 6 | 414 | BICR.18 | Head and Neck cancer |
| 713 | 6 | 189 | MKN1 | Gastric cancer |
| 714 | 6 | 851 | TE.8 | Esophageal cancer |
| 715 | 6 | 303 | A.253 | Head and Neck cancer |
| 716 | 6 | 86 | EPLC.272H | Lung cancer |
| 717 | 6 | 403 | PE.CA.PJ49 | Head and Neck cancer |
| 718 | 6 | 984 | SU.86.86 | Pancreatic cancer |
| 719 | 6 | 179 | Panc.02.13 | Pancreatic cancer |
| 720 | 6 | 590 | NCI.H2444 | Lung cancer |
| 721 | 6 | 553 | SNU.1066 | Head and Neck cancer |
| 722 | 6 | 213 | MKN7 | Gastric cancer |
| 723 | 6 | 437 | NCI.H2342 | Lung cancer |
| 724 | 6 | 735 | HCC1806 | Breast cancer |
| 725 | 6 | 845 | BICR.31 | Head and Neck cancer |
| 726 | 6 | 938 | DOK | Head and Neck cancer |
| 727 | 6 | 979 | BFTC.905 | Bladder cancer |
| 728 | 6 | 575 | SCC.4 | Head and Neck cancer |
| 729 | 6 | 395 | KYSE.520 | Esophageal cancer |
| 730 | 6 | 941 | HCC2814 | Lung cancer |
| 731 | 6 | 1168 | L3.3 | Pancreatic cancer |
| 732 | 6 | 1177 | SNU.46 | Head and Neck cancer |
| 733 | 6 | 951 | HDQ.P1 | Breast cancer |
| 734 | 6 | 459 | CAL.85.1 | Breast cancer |
| 735 | 6 | 1108 | HCC70 | Breast cancer |
| 736 | 6 | 45 | COR.L23 | Lung cancer |
| 737 | 6 | 228 | SNU.638 | Gastric cancer |
| 738 | 6 | 294 | SiHa | Cervical cancer |
| 739 | 6 | 456 | PK.59 | Pancreatic cancer |
| 740 | 6 | 1088 | HCC1954 | Breast cancer |
| 741 | 6 | 54 | SF767 | Cervical cancer |
| 742 | 6 | 1050 | ME.180 | Cervical cancer |
| 743 | 6 | 286 | LS123 | Colorectal cancer |
| 744 | 6 | 568 | HaCaT | Non-cancerous |
| 745 | 6 | 595 | HSC.4 | Head and Neck cancer |
| 746 | 6 | 295 | BHY | Head and Neck cancer |
| 747 | 6 | 391 | SNU.1076 | Head and Neck cancer |
| 748 | 6 | 787 | SNU.1041 | Head and Neck cancer |
| 749 | 6 | 1196 | PK.8 | Pancreatic cancer |
| 750 | 6 | 145 | PE.CA.PJ34..clone.C12 | Head and Neck cancer |
| 751 | 6 | 466 | SNU.1214 | Head and Neck cancer |
| 752 | 6 | 913 | BICR.16 | Head and Neck cancer |
| 753 | 6 | 70 | SCC.25 | Head and Neck cancer |
| 754 | 6 | 413 | SCC.15 | Head and Neck cancer |
| 755 | 7 | 603 | Lu.65 | Lung cancer |
| 756 | 7 | 140 | HeLa | Cervical cancer |
| 757 | 7 | 649 | NCI.H920 | Lung cancer |
| 758 | 7 | 327 | COLO.679 | Skin cancer |
| 759 | 7 | 940 | SNU.685 | Uterine cancer |
| 760 | 7 | 472 | KU.19.19 | Bladder cancer |
| 761 | 7 | 854 | DU145 | Prostate cancer |
| 762 | 7 | 126 | NCI.H1651 | Lung cancer |
| 763 | 7 | 610 | Li.7 | Liver cancer |
| 764 | 7 | 230 | PLC.PRF.5 | Liver cancer |
| 765 | 7 | 721 | X8.MG.BA | #N/A |
| 766 | 7 | 214 | SNU.1077 | Uterine cancer |
| 767 | 7 | 220 | SNU.119 | Ovarian cancer |
| 768 | 7 | 97 | OVMANA | Ovarian cancer |
| 769 | 7 | 530 | OVISE | Ovarian cancer |
| 770 | 7 | 170 | Ishikawa..Heraklio..02.ER | Uterine cancer |
| 771 | 7 | 217 | HEC.59 | Uterine cancer |
| 772 | 7 | 79 | JHUEM.7 | Uterine cancer |
| 773 | 7 | 458 | EFE.184 | Uterine cancer |
| 774 | 7 | 707 | RMG.I | Ovarian cancer |
| 775 | 7 | 978 | HEC.151 | Uterine cancer |
| 776 | 7 | 260 | A.549 | Lung cancer |
| 777 | 7 | 481 | OAW42 | Ovarian cancer |
| 778 | 7 | 383 | GTL.16 | Gastric cancer |
| 779 | 7 | 1003 | MKN45 | Gastric cancer |
| 780 | 7 | 71 | IGROV.1 | Ovarian cancer |
| 781 | 7 | 513 | CAL.51 | Breast cancer |
| 782 | 7 | 534 | HEC.265 | Uterine cancer |
| 783 | 7 | 711 | HEC.6 | Uterine cancer |
| 784 | 7 | 68 | HEC.1.A | Uterine cancer |
| 785 | 7 | 325 | TEN | Uterine cancer |
| 786 | 7 | 423 | HEC.1.B | Uterine cancer |
| 787 | 7 | 730 | HEC.251 | Uterine cancer |
| 788 | 7 | 450 | HCC15 | Lung cancer |
| 789 | 7 | 547 | SNG.M | Uterine cancer |
| 790 | 7 | 251 | Panc.10.05 | Pancreatic cancer |
| 791 | 7 | 440 | PL45 | Pancreatic cancer |
| 792 | 7 | 686 | Sq.1 | Lung cancer |
| 793 | 7 | 1125 | KP.3 | Pancreatic cancer |
| 794 | 7 | 308 | HCT.15 | Colorectal cancer |
| 795 | 7 | 1184 | HCT.116 | Colorectal cancer |
| 796 | 7 | 259 | KM12 | Colorectal cancer |
| 797 | 7 | 963 | SNU.C4 | Colorectal cancer |
| 798 | 7 | 101 | SNU.5 | Gastric cancer |
| 799 | 7 | 579 | Okajima | Gastric cancer |
| 800 | 7 | 886 | NCI.H1435 | Lung cancer |
| 801 | 7 | 931 | NCI.H1573 | Lung cancer |
| 802 | 7 | 235 | SNU.869 | Bile duct cancer |
| 803 | 7 | 1104 | SW620 | Colorectal cancer |
| 804 | 7 | 116 | SNU.407 | Colorectal cancer |
| 805 | 7 | 855 | HCA7 | Colorectal cancer |
| 806 | 7 | 550 | SNU.81 | Colorectal cancer |
| 807 | 7 | 328 | LoVo | Colorectal cancer |
| 808 | 7 | 883 | SNU.1040 | Colorectal cancer |
| 809 | 7 | 810 | LN.308 | Brain cancer |
| 810 | 7 | 421 | X253J.BV | #N/A |
| 811 | 7 | 796 | X253J | #N/A |
| 812 | 7 | 1164 | SNU.349 | Kidney cancer |
| 813 | 7 | 573 | HCC1319 | Kidney cancer |
| 814 | 7 | 887 | KMRC.3 | Kidney cancer |
| 815 | 7 | 442 | VMRC.RCW | Kidney cancer |
| 816 | 7 | 364 | SLR23 | Kidney cancer |
| 817 | 7 | 1064 | KMRC.2 | Kidney cancer |
| 818 | 7 | 484 | KMRC.20 | Kidney cancer |
| 819 | 7 | 562 | RCC10RGB | Kidney cancer |
| 820 | 7 | 933 | SNU.1272 | Kidney cancer |
| 821 | 7 | 1175 | TUHR10TKB | Kidney cancer |
| 822 | 7 | 347 | TUHR4TKB | Kidney cancer |
| 823 | 7 | 366 | SW1710 | Bladder cancer |
| 824 | 7 | 695 | TK.10 | Kidney cancer |
| 825 | 7 | 952 | HEC.50B | Uterine cancer |
| 826 | 7 | 162 | HCC1011 | Kidney cancer |
| 827 | 7 | 455 | A.498 | Kidney cancer |
| 828 | 7 | 1075 | Caki.2 | Kidney cancer |
| 829 | 7 | 278 | ACHN | Kidney cancer |
| 830 | 7 | 1154 | CAL.54 | Kidney cancer |
| 831 | 7 | 903 | SNU.1079 | Bile duct cancer |
| 832 | 7 | 55 | VMRC.RCZ | Kidney cancer |
| 833 | 7 | 376 | TUHR14TKB | Kidney cancer |
| 834 | 7 | 785 | SLR21 | Kidney cancer |
| 835 | 7 | 330 | SLR26 | Kidney cancer |
| 836 | 7 | 793 | NCI.H28 | Lung cancer |
| 837 | 7 | 1099 | X786.O | #N/A |
| 838 | 7 | 297 | X769.P | #N/A |
| 839 | 7 | 694 | A.704 | Kidney cancer |
| 840 | 7 | 1059 | HCC89 | Kidney cancer |
| 841 | 7 | 1092 | RPTEC.TERT1 | Non-cancerous |
| 842 | 7 | 1144 | OELE | Non-cancerous |
| 843 | 7 | 929 | SNU.449 | Liver cancer |
| 844 | 7 | 293 | TOV.21G | Ovarian cancer |
| 845 | 7 | 821 | OVTOKO | Ovarian cancer |
| 846 | 7 | 778 | NCI.H1944 | Lung cancer |
| 847 | 7 | 121 | SNU.878 | Liver cancer |
| 848 | 7 | 188 | HuH.1 | Liver cancer |
| 849 | 7 | 427 | JHH.5 | Liver cancer |
| 850 | 7 | 629 | OV.90 | Ovarian cancer |
| 851 | 7 | 606 | Fu97 | Gastric cancer |
| 852 | 7 | 401 | Hep.3B2.1.7 | Liver cancer |
| 853 | 7 | 535 | Huh.7 | Liver cancer |
| 854 | 7 | 558 | JHH.7 | Liver cancer |
| 855 | 7 | 607 | Hep.G2 | Liver cancer |
| 856 | 7 | 1201 | HuH.6 | Liver cancer |
| 857 | 7 | 12 | C2BBe1 | Colorectal cancer |
| 858 | 7 | 708 | CACO.2 | Colorectal cancer |
| 859 | 7 | 265 | SNU.620 | Gastric cancer |
| 860 | 7 | 299 | LS411N | Colorectal cancer |
| 861 | 7 | 373 | HCC1263 | Colorectal cancer |
| 862 | 7 | 334 | SNU.C1 | Colorectal cancer |
| 863 | 7 | 379 | SNU.719 | Gastric cancer |
| 864 | 7 | 593 | NCI.H854 | Lung cancer |
| 865 | 7 | 985 | CL.40 | Colorectal cancer |
| 866 | 7 | 619 | HCC1588 | Lung cancer |
| 867 | 7 | 916 | LS513 | Colorectal cancer |
| 868 | 7 | 123 | GCIY | Gastric cancer |
| 869 | 7 | 531 | KATO.III | Gastric cancer |
| 870 | 7 | 693 | IM95 | Gastric cancer |
| 871 | 7 | 800 | SNU.520 | Gastric cancer |
| 872 | 7 | 944 | SNU.16 | Gastric cancer |
| 873 | 7 | 1043 | PaTu.8988s | Pancreatic cancer |
| 874 | 7 | 409 | GSU | Gastric cancer |
| 875 | 7 | 749 | HuG1.N | Gastric cancer |
| 876 | 7 | 387 | OCUM.1 | Gastric cancer |
| 877 | 7 | 654 | NUGC.4 | Gastric cancer |
| 878 | 7 | 598 | CW.2 | Colorectal cancer |
| 879 | 7 | 744 | JHOM.2B | Ovarian cancer |
| 880 | 7 | 232 | RCM.1..Human.rectal | Colorectal cancer |
| 881 | 7 | 797 | CL.34 | Colorectal cancer |
| 882 | 7 | 441 | HT115 | Colorectal cancer |
| 883 | 7 | 446 | SNU.61 | Colorectal cancer |
| 884 | 7 | 1166 | T84 | Colorectal cancer |
| 885 | 7 | 488 | SW948 | Colorectal cancer |
| 886 | 7 | 538 | NCI.H508 | Colorectal cancer |
| 887 | 7 | 647 | CCK.81 | Colorectal cancer |
| 888 | 7 | 227 | LS180 | Colorectal cancer |
| 889 | 7 | 1037 | GP2d | Colorectal cancer |
| 890 | 7 | 618 | NCI.H684 | Colorectal cancer |
| 891 | 7 | 180 | HT.55 | Colorectal cancer |
| 892 | 7 | 750 | SNU.283 | Colorectal cancer |
| 893 | 7 | 313 | HCC.56 | Colorectal cancer |
| 894 | 7 | 284 | SW403 | Colorectal cancer |
| 895 | 7 | 921 | SW1463 | Colorectal cancer |
| 896 | 7 | 542 | SNU.245 | Bile duct cancer |
| 897 | 7 | 791 | HCC630 | Ovarian cancer |
| 898 | 7 | 563 | SNU.1033 | Colorectal cancer |
| 899 | 7 | 600 | CL.14 | Colorectal cancer |
| 900 | 7 | 248 | SW1116 | Colorectal cancer |
| 901 | 7 | 876 | LS1034 | Colorectal cancer |
| 902 | 7 | 273 | OE19 | Esophageal cancer |
| 903 | 7 | 1105 | SK.CO.1 | Colorectal cancer |
| 904 | 7 | 182 | COLO.201 | Colorectal cancer |
| 905 | 7 | 661 | COLO.206F | Colorectal cancer |
| 906 | 7 | 58 | KMS.11 | Myeloma |
| 907 | 7 | 898 | KMS.28BM | Myeloma |
| 908 | 7 | 83 | SNU.478 | Bile duct cancer |
| 909 | 7 | 175 | SNU.761 | Liver cancer |
| 910 | 7 | 342 | JHH.2 | Liver cancer |
| 911 | 7 | 290 | KYSE.510 | Esophageal cancer |
| 912 | 7 | 495 | HCC4011 | Lung cancer |
| 913 | 7 | 527 | HCC4006 | Lung cancer |
| 914 | 7 | 215 | Panc.03.27 | Pancreatic cancer |
| 915 | 7 | 804 | NCI.H2073 | Lung cancer |
| 916 | 7 | 476 | SNU.324 | Pancreatic cancer |
| 917 | 7 | 660 | HCC364 | Lung cancer |
| 918 | 7 | 152 | T3M.10 | Lung cancer |
| 919 | 7 | 138 | SUIT.2 | Pancreatic cancer |
| 920 | 7 | 1124 | NCI.H2887 | Lung cancer |
| 921 | 8 | 474 | WSU.FSCCL | Lymphoma |
| 922 | 8 | 505 | TK..Human.B.cell | Lymphoma |
| 923 | 8 | 453 | DB | Lymphoma |
| 924 | 8 | 310 | HT | Lymphoma |
| 925 | 8 | 312 | Karpas.422 | Lymphoma |
| 926 | 8 | 811 | MHH.PREB.1 | Lymphoma |
| 927 | 8 | 41 | Sc.1 | Lymphoma |
| 928 | 8 | 525 | JM.1 | Leukemia |
| 929 | 8 | 682 | Toledo | Lymphoma |
| 930 | 8 | 545 | GA.10 | Lymphoma |
| 931 | 8 | 1044 | BL.41 | Lymphoma |
| 932 | 8 | 991 | BL.70 | Lymphoma |
| 933 | 8 | 1072 | Ramos | Lymphoma |
| 934 | 8 | 1181 | KMS.26 | Myeloma |
| 935 | 8 | 823 | JJN.3 | Myeloma |
| 936 | 8 | 154 | KMS.34 | Myeloma |
| 937 | 8 | 577 | MM1.S | Myeloma |
| 938 | 8 | 588 | LP.1 | Myeloma |
| 939 | 8 | 56 | KMS.27 | Myeloma |
| 940 | 8 | 880 | L.363 | Myeloma |
| 941 | 8 | 641 | U.266.70 | Myeloma |
| 942 | 8 | 207 | INA.6 | Myeloma |
| 943 | 8 | 758 | NCI.H929 | Myeloma |
| 944 | 8 | 642 | OCI.My5 | Myeloma |
| 945 | 8 | 428 | OPM.2 | Myeloma |
| 946 | 8 | 1000 | OPM.1 | Myeloma |
| 947 | 8 | 1031 | KMS.18 | Myeloma |
| 948 | 8 | 275 | U.266.84 | Myeloma |
| 949 | 8 | 671 | U266B1 | Myeloma |
| 950 | 8 | 332 | MOLP.2 | Myeloma |
| 951 | 8 | 639 | KMS.12.BM | Myeloma |
| 952 | 8 | 504 | Karpas.620 | Myeloma |
| 953 | 8 | 515 | KMS.20 | Myeloma |
| 954 | 8 | 115 | SK.MM.2 | Myeloma |
| 955 | 8 | 1083 | EJM | Myeloma |
| 956 | 8 | 141 | AMO1 | Myeloma |
| 957 | 8 | 143 | KHM.1B | Myeloma |
| 958 | 8 | 151 | A3.Kawakami | Lymphoma |
| 959 | 8 | 223 | OCI.My7 | Myeloma |
| 960 | 8 | 628 | A4.Fukuda | Lymphoma |
| 961 | 8 | 1068 | KMS.21.BM | Myeloma |
| 962 | 8 | 1150 | MOLP.8 | Myeloma |
| 963 | 8 | 852 | OCI.M2 | Leukemia |
| 964 | 8 | 943 | JURL.MK1 | Leukemia |
| 965 | 8 | 410 | KYO.1 | Leukemia |
| 966 | 8 | 127 | K.562 | Leukemia |
| 967 | 8 | 565 | HMC.1 | Leukemia |
| 968 | 8 | 142 | F.36P | Leukemia |
| 969 | 8 | 718 | OCI.M1 | Leukemia |
| 970 | 8 | 378 | JK.1 | Leukemia |
| 971 | 8 | 1111 | LAMA.84 | Leukemia |
| 972 | 8 | 723 | HEL.92.1.7 | Leukemia |
| 973 | 8 | 1012 | HEL | Leukemia |
| 974 | 8 | 221 | CMK | Leukemia |
| 975 | 8 | 204 | MOLM.16 | Leukemia |
| 976 | 8 | 965 | SET.2 | Leukemia |
| 977 | 8 | 807 | NALM.1 | Leukemia |
| 978 | 8 | 354 | MHH.CALL.4 | Leukemia |
| 979 | 8 | 578 | MHH.CALL.2 | Leukemia |
| 980 | 8 | 634 | SUP.B15 | Leukemia |
| 981 | 8 | 884 | Kasumi.2 | Leukemia |
| 982 | 8 | 1078 | BV.173 | Leukemia |
| 983 | 8 | 549 | MHH.CALL.3 | Leukemia |
| 984 | 8 | 1006 | NALM.6 | Leukemia |
| 985 | 8 | 426 | NALM.19 | Leukemia |
| 986 | 8 | 1182 | MUTZ.5 | Leukemia |
| 987 | 8 | 306 | RS4.11 | #N/A |
| 988 | 8 | 317 | SEM | Leukemia |
| 989 | 8 | 158 | KOPN.8 | Leukemia |
| 990 | 8 | 878 | REH | Leukemia |
| 991 | 8 | 953 | X697 | #N/A |
| 992 | 8 | 1187 | OCI.Ly19 | Lymphoma |
| 993 | 8 | 222 | KCL.22 | Leukemia |
| 994 | 8 | 849 | RCH.ACV | Leukemia |
| 995 | 8 | 701 | UKE.1 | Leukemia |
| 996 | 8 | 969 | ME.1..Human.leukemia. | Leukemia |
| 997 | 8 | 1191 | NCO2 | Leukemia |
| 998 | 8 | 1005 | HNT.34 | Leukemia |
| 999 | 8 | 512 | MOLM.6 | Leukemia |
| 1000 | 8 | 927 | KG.1 | Leukemia |
| 1001 | 8 | 382 | Loucy | Leukemia |
| 1002 | 8 | 585 | P31.FUJ | Leukemia |
| 1003 | 8 | 324 | JURKAT | Leukemia |
| 1004 | 8 | 751 | Jurkat.E6.1 | Leukemia |
| 1005 | 8 | 541 | CML.T1 | Leukemia |
| 1006 | 8 | 1015 | Peer | Leukemia |
| 1007 | 8 | 349 | HPB.ALL | Leukemia |
| 1008 | 8 | 245 | PF.382 | Leukemia |
| 1009 | 8 | 1186 | KE.37 | Leukemia |
| 1010 | 8 | 982 | MOLT.13 | Leukemia |
| 1011 | 8 | 1159 | P12.Ichikawa | Leukemia |
| 1012 | 8 | 202 | MOLT.3 | Leukemia |
| 1013 | 8 | 789 | MOLT.16 | Leukemia |
| 1014 | 8 | 1165 | TALL.1..Human.adult.T | Leukemia |
| 1015 | 8 | 714 | MOLT.4 | Leukemia |
| 1016 | 8 | 799 | RPMI.8402 | Leukemia |
| 1017 | 8 | 1176 | ALL.SIL | Leukemia |
| 1018 | 8 | 637 | TF.1 | Leukemia |
| 1019 | 8 | 161 | M.07e | Leukemia |
| 1020 | 8 | 974 | Ku812 | Leukemia |
| 1021 | 8 | 323 | SKNO.1 | Leukemia |
| 1022 | 8 | 798 | PL.21 | Leukemia |
| 1023 | 8 | 490 | PLB.985 | Leukemia |
| 1024 | 8 | 1084 | HL.60 | Leukemia |
| 1025 | 8 | 105 | U.937 | Leukemia |
| 1026 | 8 | 448 | Kasumi.1 | Leukemia |
| 1027 | 8 | 975 | EM.2 | Leukemia |
| 1028 | 8 | 1129 | AML.193 | Leukemia |
| 1029 | 8 | 443 | Kasumi.6 | Leukemia |
| 1030 | 8 | 1062 | KO52 | Leukemia |
| 1031 | 8 | 185 | MUTZ.3 | Leukemia |
| 1032 | 8 | 1149 | GDM.1 | Leukemia |
| 1033 | 8 | 698 | OCI.AML.3 | Leukemia |
| 1034 | 8 | 90 | SIG.M5 | Leukemia |
| 1035 | 8 | 626 | OCI.AML.5 | Leukemia |
| 1036 | 8 | 684 | THP.1 | Leukemia |
| 1037 | 8 | 331 | SKM.1 | Leukemia |
| 1038 | 8 | 1115 | NOMO.1 | Leukemia |
| 1039 | 8 | 100 | NB4 | Leukemia |
| 1040 | 8 | 492 | MOLM.13 | Leukemia |
| 1041 | 8 | 794 | EoL.1 | Leukemia |
| 1042 | 8 | 475 | Mono.Mac.1 | Leukemia |
| 1043 | 8 | 604 | Mono.Mac.6 | Leukemia |
| 1044 | 8 | 1171 | ML.2 | Leukemia |
| 1045 | 8 | 65 | OCI.AML.2 | Leukemia |
| 1046 | 8 | 1202 | MV4.11 | Leukemia |
| 1047 | 8 | 419 | Karpas.707 | Myeloma |
| 1048 | 8 | 748 | Ki.JK | Lymphoma |
| 1049 | 8 | 37 | HuT.102 | Lymphoma |
| 1050 | 8 | 627 | DEL | Lymphoma |
| 1051 | 8 | 159 | SR | Lymphoma |
| 1052 | 8 | 355 | SUP.M2 | Lymphoma |
| 1053 | 8 | 146 | KM.H2 | Lymphoma |
| 1054 | 8 | 769 | RC.K8 | Lymphoma |
| 1055 | 8 | 187 | HuNS1 | Myeloma |
| 1056 | 8 | 40 | JVM.3 | Leukemia |
| 1057 | 8 | 1056 | Jiyoye | Lymphoma |
| 1058 | 8 | 1087 | EHEB | Lymphoma |
| 1059 | 8 | 901 | JVM.2 | Lymphoma |
| 1060 | 8 | 743 | CCRF.SB | Leukemia |
| 1061 | 8 | 514 | BDCM | Leukemia |
| 1062 | 8 | 1142 | COR.L26 | Lung cancer |
| 1063 | 8 | 157 | C8166 | Lymphoma |
| 1064 | 8 | 692 | MJ | Lymphoma |
| 1065 | 8 | 987 | L.1236 | Lymphoma |
| 1066 | 8 | 172 | HuT.78 | Lymphoma |
| 1067 | 8 | 872 | HDLM.2 | Lymphoma |
| 1068 | 8 | 667 | Pfeiffer | Lymphoma |
| 1069 | 8 | 981 | JeKo.1 | Lymphoma |
| 1070 | 8 | 167 | Farage | Lymphoma |
| 1071 | 8 | 1020 | REC.1 | Lymphoma |
| 1072 | 8 | 280 | OCI.Ly3 | Lymphoma |
| 1073 | 8 | 896 | TMD8 | Lymphoma |
| 1074 | 8 | 838 | NU.DHL.1 | Lymphoma |
| 1075 | 8 | 875 | OCI.Ly10 | Lymphoma |
| 1076 | 8 | 732 | KE.97 | Myeloma |
| 1077 | 8 | 362 | RL | Lymphoma |
| 1078 | 8 | 1169 | SU.DHL.10 | Lymphoma |
| 1079 | 8 | 973 | Daudi | Lymphoma |
| 1080 | 8 | 1 | CA46 | Lymphoma |
| 1081 | 8 | 777 | SU.DHL.4 | Lymphoma |
| 1082 | 8 | 250 | P3HR.1 | Lymphoma |
| 1083 | 8 | 621 | DoHH2 | Lymphoma |
| 1084 | 8 | 1040 | Hs.611.T | Lymphoma |
| 1085 | 8 | 147 | WSU.NHL | Lymphoma |
| 1086 | 8 | 1034 | U.2932 | Lymphoma |
| 1087 | 8 | 710 | Raji | Lymphoma |
| 1088 | 8 | 69 | SU.DHL.5 | Lymphoma |
| 1089 | 8 | 52 | OCI.Ly7 | Lymphoma |
| 1090 | 8 | 269 | SU.DHL.6 | Lymphoma |
| 1091 | 8 | 645 | WSU.DLCL2 | Lymphoma |
| 1092 | 8 | 722 | BJAB | Lymphoma |
| 1093 | 8 | 926 | U.698.M | Lymphoma |
| 1094 | 8 | 685 | Mino | Lymphoma |
| 1095 | 8 | 830 | Namalwa | Lymphoma |
| 1096 | 8 | 1101 | Ri.1 | Lymphoma |
| 1097 | 8 | 687 | EB2 | Lymphoma |
| 1098 | 8 | 776 | EB1 | Lymphoma |
| 1099 | 8 | 33 | MEC.1 | Leukemia |
| 1100 | 8 | 808 | Granta.519 | Lymphoma |
| 1101 | 8 | 803 | NU.DUL.1 | Lymphoma |
| 1102 | 8 | 13 | Karpas.1106P | Lymphoma |
| 1103 | 8 | 679 | SU.DHL.8 | Lymphoma |
| 1104 | 8 | 1199 | Ci.1 | Lymphoma |
| 1105 | 8 | 81 | SU.DHL.16 | Lymphoma |
| 1106 | 8 | 552 | EB3..Human.Burkitt | Lymphoma |
| 1107 | 8 | 930 | MC116 | Lymphoma |
| 1108 | 8 | 44 | L.540 | Lymphoma |
| 1109 | 8 | 591 | L.428 | Lymphoma |
| 1110 | 8 | 209 | HH..Human.lymphoma. | Lymphoma |
| 1111 | 8 | 249 | SUP.T11 | Leukemia |
| 1112 | 8 | 171 | MEG.01 | Leukemia |
| 1113 | 8 | 648 | SUP.T1 | Leukemia |
| 1114 | 8 | 726 | DND.41 | Leukemia |
| 1115 | 8 | 1180 | ST486 | Lymphoma |
| 1116 | 8 | 186 | BCP.1 | Lymphoma |
| 1117 | 8 | 352 | KMM.1 | Myeloma |
| 1118 | 9 | 1013 | NCI.N87 | Gastric cancer |
| 1119 | 9 | 380 | KCI.MOH1 | Pancreatic cancer |
| 1120 | 9 | 839 | PaTu.8902 | Pancreatic cancer |
| 1121 | 9 | 343 | OVCAR.5 | Ovarian cancer |
| 1122 | 9 | 846 | HuP.T3 | Pancreatic cancer |
| 1123 | 9 | 224 | CAPAN.2 | Pancreatic cancer |
| 1124 | 9 | 1135 | Panc.04.03 | Pancreatic cancer |
| 1125 | 9 | 62 | T3M.4 | Pancreatic cancer |
| 1126 | 9 | 741 | NCI.H1666 | Lung cancer |
| 1127 | 9 | 836 | PE.CA.PJ15 | Head and Neck cancer |
| 1128 | 9 | 1016 | RMUG.S | Ovarian cancer |
| 1129 | 9 | 47 | NCI.H2126 | Lung cancer |
| 1130 | 9 | 1109 | RERF.LC.KJ | Lung cancer |
| 1131 | 9 | 508 | NUGC.3 | Gastric cancer |
| 1132 | 9 | 608 | HuCC.T1 | Bile duct cancer |
| 1133 | 9 | 819 | AGS | Gastric cancer |
| 1134 | 9 | 67 | SNU.601 | Gastric cancer |
| 1135 | 9 | 135 | KE.39 | Gastric cancer |
| 1136 | 9 | 113 | X23132.87 | #N/A |
| 1137 | 9 | 928 | SNU.175 | Colorectal cancer |
| 1138 | 9 | 998 | TCC.Pan2 | Pancreatic cancer |
| 1139 | 9 | 1009 | AsPC.1 | Pancreatic cancer |
| 1140 | 9 | 20 | Capan.1 | Pancreatic cancer |
| 1141 | 9 | 309 | HT.29 | Colorectal cancer |
| 1142 | 9 | 526 | HPAF.II | Pancreatic cancer |
| 1143 | 9 | 977 | Panc.05.04 | Pancreatic cancer |
| 1144 | 9 | 1128 | HuP.T4 | Pancreatic cancer |
| 1145 | 9 | 393 | CFPAC.1 | Pancreatic cancer |
| 1146 | 9 | 958 | Calu.3 | Lung cancer |
| 1147 | 9 | 102 | SNU.1197 | Colorectal cancer |
| 1148 | 9 | 1151 | C170 | Colorectal cancer |
| 1149 | 9 | 857 | MCAS | Ovarian cancer |
| 1150 | 9 | 633 | Panc.08.13 | Pancreatic cancer |
| 1151 | 9 | 1116 | Panc.02.03 | Pancreatic cancer |
| 1152 | 9 | 1167 | SNU.213 | Pancreatic cancer |
| 1153 | 9 | 809 | DAN.G | Pancreatic cancer |
| 1154 | 9 | 919 | SW1990 | Pancreatic cancer |
| 1155 | 9 | 321 | HCC1493 | Breast cancer |
| 1156 | 9 | 681 | NCI.H2291 | Lung cancer |
| 1157 | 9 | 74 | NCI.H820 | Lung cancer |
| 1158 | 9 | 396 | HCC2302 | Lung cancer |
| 1159 | 9 | 956 | HCC827.GR5 | Lung cancer |
| 1160 | 9 | 605 | NCI.H1648 | Lung cancer |
| 1161 | 9 | 1107 | NCI.H2347 | Lung cancer |
| 1162 | 9 | 1086 | NCI.H1373 | Lung cancer |
| 1163 | 9 | 1002 | NCI.H441 | Lung cancer |
| 1164 | 9 | 1048 | HCC78 | Lung cancer |
| 1165 | 9 | 49 | CAL.12T | Lung cancer |
| 1166 | 9 | 150 | JH.EsoAd1 | Esophageal cancer |
| 1167 | 9 | 184 | PE.CA.PJ41..clone.D2 | Head and Neck cancer |
| 1168 | 9 | 1143 | RERF.LC.Ad2 | Lung cancer |
| 1169 | 9 | 398 | PK.1 | Pancreatic cancer |
| 1170 | 9 | 960 | X647V | #N/A |
| 1171 | 9 | 23 | CaR.1 | Colorectal cancer |
| 1172 | 9 | 742 | X5637 | #N/A |
| 1173 | 9 | 89 | SUM52PE | Breast cancer |
| 1174 | 9 | 895 | SNU.8 | Ovarian cancer |
| 1175 | 9 | 1069 | YAPC | Pancreatic cancer |
| 1176 | 9 | 229 | RERF.GC.1B | Gastric cancer |
| 1177 | 9 | 1046 | NCC.StC.K140 | Gastric cancer |
| 1178 | 9 | 580 | HT.1376 | Bladder cancer |
| 1179 | 9 | 1117 | HT.1197 | Bladder cancer |
| 1180 | 9 | 537 | BICR.6 | Head and Neck cancer |
| 1181 | 9 | 899 | BICR.78 | Head and Neck cancer |
| 1182 | 9 | 258 | BICR.56 | Head and Neck cancer |
| 1183 | 9 | 889 | SCC.9 | Head and Neck cancer |
| 1184 | 9 | 94 | UACC.812 | Breast cancer |
| 1185 | 9 | 196 | BEWO | Uncategorized |
| 1186 | 9 | 500 | OUMS.23 | Colorectal cancer |
| 1187 | 9 | 615 | HN | Head and Neck cancer |
| 1188 | 9 | 757 | CL.11 | Colorectal cancer |
| 1189 | 9 | 995 | SW1417 | Colorectal cancer |
| 1190 | 9 | 1102 | VM.CUB.1 | Bladder cancer |
| 1191 | 9 | 212 | YD.38 | Head and Neck cancer |
| 1192 | 9 | 1070 | YD.15 | Head and Neck cancer |
| 1193 | 9 | 163 | SNU.C2A | Colorectal cancer |
| 1194 | 9 | 1080 | SW48 | Colorectal cancer |
| 1195 | 9 | 589 | SNU.C5 | Colorectal cancer |
| 1196 | 9 | 818 | TGBC11TKB | Gastric cancer |
| 1197 | 9 | 8 | HCC1143 | Breast cancer |
| 1198 | 9 | 658 | HCC1937 | Breast cancer |
| 1199 | 9 | 709 | COV644 | Ovarian cancer |
| 1200 | 9 | 1057 | TE.10 | Esophageal cancer |
| 1201 | 9 | 341 | TE.6 | Esophageal cancer |
| 1202 | 9 | 942 | TE.14 | Esophageal cancer |
| 1203 | 9 | 662 | U.BLC1 | Bladder cancer |
| 1204 | 9 | 164 | BxPC.3 | Pancreatic cancer |
| 1205 | 9 | 301 | KMBC.2 | Bladder cancer |
| 1206 | 9 | 516 | UM.UC.1 | Bladder cancer |

1. **Row (transcription factor) information of clustering results**

| **Row** | **Cluster** | **Transcription Factor Index** | **Transcription Factor Symbol** |
| --- | --- | --- | --- |
| 1 | 1 | 168 | BACH1 |
| 2 | 1 | 460 | AHR |
| 3 | 1 | 187 | FOXO6 |
| 4 | 1 | 488 | RXRG |
| 5 | 1 | 71 | XBP1 |
| 6 | 1 | 436 | ZBED1 |
| 7 | 1 | 25 | TBP |
| 8 | 1 | 346 | ZNF343 |
| 9 | 1 | 372 | ZNF449 |
| 10 | 1 | 452 | ETV7 |
| 11 | 1 | 14 | ETV5 |
| 12 | 1 | 206 | FEV |
| 13 | 1 | 103 | FLI1 |
| 14 | 1 | 196 | ERF |
| 15 | 1 | 149 | ETS1 |
| 16 | 1 | 195 | ETV4 |
| 17 | 1 | 202 | ZBTB7A |
| 18 | 1 | 4 | ETV1 |
| 19 | 1 | 204 | ETV6 |
| 20 | 1 | 64 | ELF4 |
| 21 | 1 | 547 | PLAGL2 |
| 22 | 1 | 450 | EGR2 |
| 23 | 1 | 98 | NFIX |
| 24 | 1 | 246 | NFIC |
| 25 | 1 | 184 | RARG |
| 26 | 1 | 422 | ZNF528 |
| 27 | 1 | 326 | MSANTD3 |
| 28 | 1 | 518 | ZBTB48 |
| 29 | 1 | 16 | RARA |
| 30 | 1 | 11 | RARB |
| 31 | 1 | 347 | IRF4 |
| 32 | 1 | 274 | ZNF121 |
| 33 | 1 | 308 | IRF1 |
| 34 | 1 | 439 | LMX1B |
| 35 | 1 | 449 | RUNX3 |
| 36 | 1 | 23 | NR4A1 |
| 37 | 1 | 146 | NR4A2 |
| 38 | 1 | 226 | ZNF684 |
| 39 | 1 | 172 | GABPA |
| 40 | 1 | 39 | RXRA |
| 41 | 1 | 216 | IRF6 |
| 42 | 2 | 287 | ATF2 |
| 43 | 2 | 335 | LIN54 |
| 44 | 2 | 99 | ZNF136 |
| 45 | 2 | 348 | YY2 |
| 46 | 2 | 393 | ZNF76 |
| 47 | 2 | 178 | YY1 |
| 48 | 2 | 201 | NRF1 |
| 49 | 2 | 35 | ELK4 |
| 50 | 2 | 117 | ELK1 |
| 51 | 2 | 194 | ELK3 |
| 52 | 2 | 54 | THRA |
| 53 | 2 | 140 | SMARCA1 |
| 54 | 2 | 442 | TCFL5 |
| 55 | 2 | 137 | PBX1 |
| 56 | 2 | 406 | HMBOX1 |
| 57 | 2 | 18 | JUND |
| 58 | 2 | 125 | JUN |
| 59 | 2 | 344 | FOSL1 |
| 60 | 2 | 212 | JUNB |
| 61 | 2 | 156 | FOS |
| 62 | 2 | 55 | FOSL2 |
| 63 | 2 | 124 | FOSB |
| 64 | 2 | 68 | ZNF317 |
| 65 | 2 | 122 | MECOM |
| 66 | 2 | 126 | MAX |
| 67 | 2 | 390 | MNT |
| 68 | 2 | 472 | ASCL1 |
| 69 | 2 | 536 | ZNF418 |
| 70 | 3 | 79 | RFX5 |
| 71 | 3 | 88 | RFX3 |
| 72 | 3 | 113 | RFX2 |
| 73 | 3 | 224 | KLF1 |
| 74 | 3 | 169 | KLF12 |
| 75 | 3 | 534 | KLF7 |
| 76 | 3 | 499 | PATZ1 |
| 77 | 3 | 362 | SP1 |
| 78 | 3 | 189 | SP2 |
| 79 | 3 | 139 | KLF10 |
| 80 | 3 | 319 | SP4 |
| 81 | 3 | 419 | GFI1 |
| 82 | 3 | 177 | MYBL1 |
| 83 | 3 | 293 | SATB1 |
| 84 | 3 | 356 | ZFP28 |
| 85 | 3 | 529 | ASCL2 |
| 86 | 3 | 286 | RELB |
| 87 | 3 | 429 | ZNF282 |
| 88 | 3 | 453 | ZFP82 |
| 89 | 3 | 303 | SIX2 |
| 90 | 3 | 145 | SIX1 |
| 91 | 3 | 544 | SIX4 |
| 92 | 3 | 78 | LHX1 |
| 93 | 3 | 84 | SOX9 |
| 94 | 3 | 385 | TFE3 |
| 95 | 3 | 551 | MLXIPL |
| 96 | 3 | 114 | LHX2 |
| 97 | 3 | 383 | KLF15 |
| 98 | 4 | 110 | STAT5A |
| 99 | 4 | 283 | STAT4 |
| 100 | 4 | 298 | STAT3 |
| 101 | 4 | 352 | ZNF260 |
| 102 | 4 | 391 | HMX1 |
| 103 | 4 | 135 | ZFP42 |
| 104 | 4 | 154 | NR2F1 |
| 105 | 4 | 148 | HSF1 |
| 106 | 4 | 218 | HSF4 |
| 107 | 4 | 107 | TAF1 |
| 108 | 4 | 90 | HIF1A |
| 109 | 4 | 157 | ARNT |
| 110 | 4 | 118 | FOXC2 |
| 111 | 4 | 121 | HOXB4 |
| 112 | 4 | 225 | FOXF2 |
| 113 | 4 | 138 | HOXA9 |
| 114 | 4 | 233 | HOXD11 |
| 115 | 4 | 458 | CREB3 |
| 116 | 4 | 474 | ZNF549 |
| 117 | 4 | 454 | TFCP2 |
| 118 | 4 | 5 | TFEC |
| 119 | 4 | 461 | SP3 |
| 120 | 4 | 100 | USF2 |
| 121 | 4 | 45 | ETS2 |
| 122 | 4 | 96 | ETV3 |
| 123 | 4 | 222 | SP8 |
| 124 | 4 | 228 | KLF16 |
| 125 | 4 | 170 | NKX6-1 |
| 126 | 4 | 355 | HOXC13 |
| 127 | 4 | 331 | FOXQ1 |
| 128 | 4 | 85 | RELA |
| 129 | 4 | 192 | REL |
| 130 | 4 | 414 | SMAD5 |
| 131 | 4 | 180 | TFDP1 |
| 132 | 4 | 261 | ATOH7 |
| 133 | 4 | 374 | ZNF423 |
| 134 | 4 | 531 | EGR3 |
| 135 | 4 | 181 | GLI2 |
| 136 | 4 | 492 | USF1 |
| 137 | 4 | 456 | RFX4 |
| 138 | 4 | 558 | VDR |
| 139 | 4 | 43 | TFAP2B |
| 140 | 4 | 376 | TFAP2C |
| 141 | 4 | 89 | ZNF143 |
| 142 | 4 | 234 | LHX6 |
| 143 | 5 | 448 | HIC1 |
| 144 | 5 | 44 | TFAP2E |
| 145 | 5 | 409 | ZBTB6 |
| 146 | 5 | 134 | RUNX2 |
| 147 | 5 | 392 | ELF2 |
| 148 | 5 | 421 | ERG |
| 149 | 5 | 151 | IKZF3 |
| 150 | 5 | 253 | ELF1 |
| 151 | 5 | 27 | PLAGL1 |
| 152 | 5 | 2 | ZBTB18 |
| 153 | 5 | 431 | HES1 |
| 154 | 5 | 361 | MLXIP |
| 155 | 5 | 495 | MYCN |
| 156 | 5 | 526 | ZSCAN22 |
| 157 | 5 | 557 | FOXL1 |
| 158 | 5 | 182 | IRF2 |
| 159 | 5 | 434 | IRF9 |
| 160 | 5 | 510 | IRF7 |
| 161 | 5 | 183 | E2F4 |
| 162 | 5 | 188 | E2F2 |
| 163 | 5 | 416 | HOXA1 |
| 164 | 5 | 478 | PROX1 |
| 165 | 5 | 290 | GLIS2 |
| 166 | 5 | 340 | NR1D2 |
| 167 | 5 | 394 | PRDM5 |
| 168 | 5 | 104 | THAP1 |
| 169 | 5 | 275 | KLF4 |
| 170 | 5 | 266 | PRRX2 |
| 171 | 5 | 22 | ARID3A |
| 172 | 5 | 129 | NFE2L1 |
| 173 | 6 | 530 | SIX3 |
| 174 | 6 | 541 | OVOL2 |
| 175 | 6 | 395 | ZNF140 |
| 176 | 6 | 400 | THRB |
| 177 | 6 | 272 | NR2F6 |
| 178 | 6 | 482 | PPARD |
| 179 | 6 | 199 | ZNF320 |
| 180 | 6 | 446 | POU4F1 |
| 181 | 6 | 152 | ZNF667 |
| 182 | 6 | 297 | DUX4 |
| 183 | 6 | 273 | FOXL2 |
| 184 | 6 | 556 | TFCP2L1 |
| 185 | 6 | 520 | MLX |
| 186 | 6 | 532 | WT1 |
| 187 | 6 | 521 | REST |
| 188 | 6 | 560 | ZNF652 |
| 189 | 6 | 469 | CDX2 |
| 190 | 6 | 158 | CDX1 |
| 191 | 6 | 418 | HOXB6 |
| 192 | 6 | 270 | ZNF8 |
| 193 | 6 | 332 | FOXO1 |
| 194 | 6 | 36 | GATA6 |
| 195 | 6 | 318 | GATA4 |
| 196 | 6 | 408 | NR5A2 |
| 197 | 6 | 86 | HNF4G |
| 198 | 6 | 164 | HNF4A |
| 199 | 6 | 405 | FOXD2 |
| 200 | 6 | 403 | ZNF490 |
| 201 | 6 | 65 | HNF1B |
| 202 | 6 | 316 | HNF1A |
| 203 | 6 | 229 | ZNF214 |
| 204 | 6 | 320 | NR1I3 |
| 205 | 6 | 398 | NR2C1 |
| 206 | 6 | 10 | TCF7L1 |
| 207 | 6 | 61 | TCF7L2 |
| 208 | 6 | 330 | PKNOX1 |
| 209 | 6 | 59 | ZNF211 |
| 210 | 6 | 91 | MAFG |
| 211 | 6 | 271 | FOXA3 |
| 212 | 6 | 425 | FOXP1 |
| 213 | 6 | 410 | ZIC1 |
| 214 | 6 | 427 | ZIC3 |
| 215 | 6 | 41 | FOXA2 |
| 216 | 6 | 105 | HEY1 |
| 217 | 6 | 350 | FOXP2 |
| 218 | 6 | 349 | FOXK1 |
| 219 | 6 | 382 | FOXO3 |
| 220 | 6 | 381 | STAT6 |
| 221 | 6 | 123 | CEBPD |
| 222 | 6 | 292 | CEBPA |
| 223 | 6 | 375 | PRRX1 |
| 224 | 6 | 555 | RAX2 |
| 225 | 6 | 179 | HOXB7 |
| 226 | 6 | 345 | HOXB8 |
| 227 | 6 | 46 | TEAD1 |
| 228 | 6 | 384 | TEAD4 |
| 229 | 6 | 388 | HESX1 |
| 230 | 6 | 371 | KLF2 |
| 231 | 6 | 221 | OVOL1 |
| 232 | 6 | 77 | TFAP2A |
| 233 | 6 | 364 | ZNF770 |
| 234 | 7 | 317 | CEBPB |
| 235 | 7 | 12 | FOXA1 |
| 236 | 7 | 278 | FOXC1 |
| 237 | 7 | 128 | MGA |
| 238 | 7 | 401 | NR1H4 |
| 239 | 7 | 341 | FOXN3 |
| 240 | 7 | 378 | ISL1 |
| 241 | 7 | 438 | SOHLH2 |
| 242 | 7 | 550 | MYC |
| 243 | 7 | 51 | MYBL2 |
| 244 | 7 | 517 | RFX7 |
| 245 | 7 | 24 | ESRRG |
| 246 | 7 | 33 | NR2F2 |
| 247 | 7 | 251 | RORA |
| 248 | 7 | 525 | NR2E3 |
| 249 | 7 | 351 | TFEB |
| 250 | 7 | 444 | BHLHE41 |
| 251 | 7 | 127 | BHLHE40 |
| 252 | 7 | 133 | BMAL1 |
| 253 | 7 | 142 | MITF |
| 254 | 7 | 167 | NFAT5 |
| 255 | 7 | 174 | NFATC2 |
| 256 | 7 | 441 | ATF1 |
| 257 | 7 | 40 | ARID3B |
| 258 | 7 | 115 | THAP11 |
| 259 | 7 | 109 | FOXM1 |
| 260 | 7 | 404 | TP63 |
| 261 | 7 | 396 | ESR2 |
| 262 | 7 | 220 | HSF2 |
| 263 | 7 | 314 | TFAP4 |
| 264 | 7 | 353 | EBF1 |
| 265 | 7 | 301 | NFE2 |
| 266 | 7 | 440 | BACH2 |
| 267 | 7 | 377 | ZNF329 |
| 268 | 7 | 185 | ARID5A |
| 269 | 7 | 130 | STAT5B |
| 270 | 7 | 321 | SRF |
| 271 | 8 | 160 | NANOG |
| 272 | 8 | 455 | ZNF416 |
| 273 | 8 | 247 | CLOCK |
| 274 | 8 | 49 | LHX3 |
| 275 | 8 | 323 | OSR2 |
| 276 | 8 | 230 | GRHL1 |
| 277 | 8 | 389 | BARX2 |
| 278 | 8 | 342 | TRPS1 |
| 279 | 8 | 354 | GATA3 |
| 280 | 8 | 20 | NR3C1 |
| 281 | 8 | 485 | NR3C2 |
| 282 | 8 | 38 | ARX |
| 283 | 8 | 282 | PAX7 |
| 284 | 8 | 223 | MEIS2 |
| 285 | 8 | 497 | PLAG1 |
| 286 | 8 | 284 | TP73 |
| 287 | 8 | 370 | OSR1 |
| 288 | 8 | 47 | NFE2L2 |
| 289 | 8 | 17 | MAF |
| 290 | 8 | 279 | AR |
| 291 | 8 | 299 | TBX6 |
| 292 | 8 | 386 | TBX18 |
| 293 | 8 | 147 | MYB |
| 294 | 8 | 561 | DUXA |
| 295 | 8 | 58 | ZNF354A |
| 296 | 8 | 219 | ZNF263 |
| 297 | 8 | 161 | HEY2 |
| 298 | 8 | 166 | ZNF41 |
| 299 | 8 | 504 | SOX4 |
| 300 | 8 | 7 | ESRRA |
| 301 | 8 | 249 | ZNF354C |
| 302 | 8 | 255 | ZNF675 |
| 303 | 8 | 241 | TEAD2 |
| 304 | 8 | 243 | TWIST1 |
| 305 | 8 | 295 | ESR1 |
| 306 | 8 | 503 | ZNF134 |
| 307 | 8 | 203 | SMARCA5 |
| 308 | 8 | 336 | GLI1 |
| 309 | 8 | 242 | PPARG |
| 310 | 8 | 252 | PPARA |
| 311 | 8 | 30 | CEBPG |
| 312 | 8 | 70 | DDIT3 |
| 313 | 8 | 136 | ATF4 |
| 314 | 8 | 159 | NFIL3 |
| 315 | 8 | 238 | TEF |
| 316 | 8 | 239 | DBP |
| 317 | 8 | 501 | NRL |
| 318 | 8 | 411 | ZNF816 |
| 319 | 8 | 423 | MAFB |
| 320 | 8 | 511 | NR1D1 |
| 321 | 8 | 554 | IRF8 |
| 322 | 8 | 94 | BATF |
| 323 | 8 | 432 | GRHL2 |
| 324 | 8 | 232 | TLX2 |
| 325 | 8 | 540 | LBX1 |
| 326 | 8 | 57 | LHX4 |
| 327 | 8 | 162 | ALX3 |
| 328 | 8 | 112 | HOXD3 |
| 329 | 8 | 559 | VSX1 |
| 330 | 8 | 132 | ALX4 |
| 331 | 8 | 373 | ALX1 |
| 332 | 8 | 357 | HOXA13 |
| 333 | 8 | 528 | HOXA2 |
| 334 | 8 | 329 | ZNF554 |
| 335 | 8 | 480 | INSM1 |
| 336 | 9 | 257 | E2F3 |
| 337 | 9 | 502 | OLIG2 |
| 338 | 9 | 227 | TWIST2 |
| 339 | 9 | 413 | BHLHE22 |
| 340 | 9 | 545 | PDX1 |
| 341 | 9 | 366 | HOXB5 |
| 342 | 9 | 369 | VAX2 |
| 343 | 9 | 564 | HOXB2 |
| 344 | 9 | 34 | TBXT |
| 345 | 9 | 477 | HMX2 |
| 346 | 9 | 542 | HMX3 |
| 347 | 9 | 235 | KLF5 |
| 348 | 9 | 281 | ZBTB14 |
| 349 | 9 | 300 | KLF3 |
| 350 | 9 | 173 | ATF6 |
| 351 | 9 | 276 | MXI1 |
| 352 | 9 | 562 | PKNOX2 |
| 353 | 9 | 205 | TGIF1 |
| 354 | 9 | 473 | TGIF2 |
| 355 | 9 | 443 | HOXD10 |
| 356 | 9 | 141 | ONECUT1 |
| 357 | 9 | 294 | ONECUT3 |
| 358 | 9 | 435 | MTF1 |
| 359 | 9 | 163 | MEIS1 |
| 360 | 9 | 359 | PBX2 |
| 361 | 9 | 13 | SNAI2 |
| 362 | 9 | 53 | ZNF257 |
| 363 | 9 | 29 | KLF6 |
| 364 | 9 | 269 | IRF3 |
| 365 | 9 | 48 | FOXJ2 |
| 366 | 9 | 467 | HIC2 |
| 367 | 9 | 508 | RREB1 |
| 368 | 9 | 451 | ZSCAN31 |
| 369 | 9 | 468 | NKX2-5 |
| 370 | 9 | 3 | TBX19 |
| 371 | 9 | 37 | NOTO |
| 372 | 9 | 210 | ZNF324 |
| 373 | 9 | 267 | CRX |
| 374 | 9 | 524 | TBX20 |
| 375 | 9 | 513 | PRDM1 |
| 376 | 9 | 527 | EMX2 |
| 377 | 9 | 120 | ZNF24 |
| 378 | 9 | 153 | SMAD4 |
| 379 | 9 | 15 | ZNF85 |
| 380 | 9 | 500 | BCL11A |
| 381 | 9 | 466 | MZF1 |
| 382 | 9 | 484 | RBPJL |
| 383 | 9 | 258 | HOXC11 |
| 384 | 9 | 407 | HOXC10 |
| 385 | 9 | 19 | ARGFX |
| 386 | 9 | 9 | HOXB3 |
| 387 | 9 | 507 | NKX6-3 |
| 388 | 9 | 546 | TBX1 |
| 389 | 9 | 52 | ZBTB26 |
| 390 | 9 | 322 | ZNF708 |
| 391 | 9 | 415 | SNAI3 |
| 392 | 9 | 28 | BATF3 |
| 393 | 9 | 1 | ZNF460 |
| 394 | 9 | 263 | ZNF784 |
| 395 | 9 | 26 | EHF |
| 396 | 9 | 31 | SPI1 |
| 397 | 9 | 42 | ELF3 |
| 398 | 9 | 457 | SPIB |
| 399 | 9 | 87 | LYL1 |
| 400 | 9 | 155 | ZNF547 |
| 401 | 9 | 69 | PAX5 |
| 402 | 9 | 32 | LBX2 |
| 403 | 9 | 363 | ZNF274 |
| 404 | 9 | 465 | TP53 |
| 405 | 9 | 186 | SOX10 |
| 406 | 9 | 459 | CUX2 |
| 407 | 9 | 358 | ARID5B |
| 408 | 9 | 83 | TCF12 |
| 409 | 9 | 334 | ZNF384 |
| 410 | 9 | 537 | ZNF135 |
| 411 | 9 | 82 | LEF1 |
| 412 | 9 | 260 | ZNF574 |
| 413 | 9 | 506 | ZNF410 |
| 414 | 9 | 498 | CREB1 |
| 415 | 9 | 538 | ZNF146 |
| 416 | 10 | 259 | ZNF680 |
| 417 | 10 | 6 | FOXJ3 |
| 418 | 10 | 208 | PRDM4 |
| 419 | 10 | 101 | HOXA11 |
| 420 | 10 | 73 | HOXC9 |
| 421 | 10 | 471 | HOXB9 |
| 422 | 10 | 491 | SOX2 |
| 423 | 10 | 494 | HOXA5 |
| 424 | 10 | 539 | FEZF1 |
| 425 | 10 | 379 | E2F7 |
| 426 | 10 | 165 | MEIS3 |
| 427 | 10 | 175 | CREB3L4 |
| 428 | 10 | 191 | NHLH1 |
| 429 | 10 | 60 | POU2F1 |
| 430 | 10 | 462 | ZNF436 |
| 431 | 10 | 76 | NR5A1 |
| 432 | 10 | 437 | GATA1 |
| 433 | 10 | 62 | GATA2 |
| 434 | 10 | 213 | TAL1 |
| 435 | 10 | 8 | HES7 |
| 436 | 10 | 445 | FOXO4 |
| 437 | 10 | 365 | NKX3-1 |
| 438 | 10 | 430 | NKX3-2 |
| 439 | 10 | 302 | HLF |
| 440 | 10 | 523 | HOXD12 |
| 441 | 10 | 72 | PITX1 |
| 442 | 10 | 75 | OTX1 |
| 443 | 10 | 426 | PHOX2A |
| 444 | 10 | 549 | NEUROD1 |
| 445 | 10 | 50 | NR1H3 |
| 446 | 10 | 289 | ZNF250 |
| 447 | 10 | 262 | CTCF |
| 448 | 10 | 397 | CTCFL |
| 449 | 10 | 248 | SMAD2 |
| 450 | 10 | 250 | TCF4 |
| 451 | 10 | 240 | SMAD3 |
| 452 | 10 | 244 | ZEB1 |
| 453 | 10 | 296 | TCF3 |
| 454 | 10 | 337 | STAT2 |
| 455 | 10 | 475 | DLX3 |
| 456 | 10 | 476 | DLX4 |
| 457 | 10 | 277 | ATF7 |
| 458 | 10 | 97 | ATF3 |
| 459 | 10 | 171 | JDP2 |
| 460 | 10 | 143 | STAT1 |
| 461 | 10 | 470 | BCL6 |
| 462 | 10 | 108 | SNAI1 |
| 463 | 10 | 285 | SREBF1 |
| 464 | 10 | 93 | KLF9 |
| 465 | 10 | 304 | FOXD3 |
| 466 | 11 | 496 | ZFX |
| 467 | 11 | 236 | DMRTA1 |
| 468 | 11 | 447 | DMRTA2 |
| 469 | 11 | 479 | ZIM3 |
| 470 | 11 | 487 | SOX8 |
| 471 | 11 | 424 | FOXH1 |
| 472 | 11 | 193 | ZBTB7C |
| 473 | 11 | 420 | ZBTB7B |
| 474 | 11 | 92 | ESRRB |
| 475 | 11 | 81 | MIXL1 |
| 476 | 11 | 207 | PTF1A |
| 477 | 11 | 313 | NFIB |
| 478 | 11 | 74 | MNX1 |
| 479 | 11 | 307 | E2F1 |
| 480 | 11 | 211 | ZBTB17 |
| 481 | 11 | 198 | CREM |
| 482 | 11 | 464 | ZNF281 |
| 483 | 11 | 95 | POU5F1 |
| 484 | 11 | 306 | ZNF582 |
| 485 | 11 | 264 | PAX6 |
| 486 | 11 | 116 | BARX1 |
| 487 | 11 | 200 | ZNF93 |
| 488 | 11 | 66 | VENTX |
| 489 | 11 | 197 | EVX2 |
| 490 | 11 | 209 | ETV2 |
| 491 | 11 | 215 | IKZF1 |
| 492 | 11 | 214 | SOX5 |
| 493 | 11 | 80 | RFX1 |
| 494 | 11 | 324 | ZNF331 |
| 495 | 11 | 483 | AIRE |
| 496 | 11 | 533 | EVX1 |
| 497 | 11 | 339 | E2F6 |
| 498 | 11 | 433 | EGR1 |
| 499 | 11 | 63 | TCF7 |
| 500 | 11 | 176 | ZNF264 |
| 501 | 11 | 486 | CUX1 |
| 502 | 11 | 237 | NKX2-1 |
| 503 | 11 | 412 | NKX2-8 |
| 504 | 11 | 481 | MAFK |
| 505 | 11 | 291 | POU6F2 |
| 506 | 11 | 515 | TCF21 |
| 507 | 11 | 519 | NFIA |
| 508 | 11 | 563 | RHOXF1 |
| 509 | 11 | 516 | CREB3L2 |
| 510 | 11 | 309 | EBF2 |
| 511 | 11 | 463 | EBF3 |
| 512 | 11 | 144 | HOXA10 |
| 513 | 11 | 305 | HOXD9 |
| 514 | 11 | 56 | PBX3 |
| 515 | 11 | 552 | FOXG1 |
| 516 | 11 | 522 | POU3F3 |
| 517 | 11 | 387 | POU3F2 |
| 518 | 11 | 67 | POU2F2 |
| 519 | 11 | 360 | POU3F1 |
| 520 | 11 | 338 | NFYB |
| 521 | 11 | 288 | NFYC |
| 522 | 11 | 333 | NFYA |
| 523 | 11 | 428 | DMRT3 |
| 524 | 11 | 190 | ZNF382 |
| 525 | 11 | 21 | ZKSCAN5 |
| 526 | 11 | 111 | POU6F1 |
| 527 | 12 | 505 | HOXA4 |
| 528 | 12 | 514 | HOXC4 |
| 529 | 12 | 328 | ZBTB33 |
| 530 | 12 | 543 | TBX3 |
| 531 | 12 | 119 | BCL6B |
| 532 | 12 | 548 | RUNX1 |
| 533 | 12 | 217 | DMRTC2 |
| 534 | 12 | 553 | DLX5 |
| 535 | 12 | 268 | ZNF341 |
| 536 | 12 | 367 | MAZ |
| 537 | 12 | 417 | ZNF467 |
| 538 | 12 | 327 | SP5 |
| 539 | 12 | 380 | VEZF1 |
| 540 | 12 | 368 | ZNF18 |
| 541 | 12 | 535 | DMBX1 |
| 542 | 12 | 254 | MAFF |
| 543 | 12 | 399 | RBPJ |
| 544 | 12 | 312 | NFATC3 |
| 545 | 12 | 102 | NFATC1 |
| 546 | 12 | 310 | NFATC4 |
| 547 | 12 | 256 | NR2C2 |
| 548 | 12 | 280 | BHLHA15 |
| 549 | 12 | 131 | SREBF2 |
| 550 | 12 | 231 | SALL4 |
| 551 | 12 | 402 | ZNF148 |
| 552 | 12 | 489 | ZNF768 |
| 553 | 12 | 490 | NFKB2 |
| 554 | 12 | 512 | PRDM14 |
| 555 | 12 | 245 | ZNF740 |
| 556 | 12 | 509 | ELF5 |
| 557 | 12 | 493 | MEF2B |
| 558 | 12 | 150 | MEF2D |
| 559 | 12 | 265 | MEF2A |
| 560 | 12 | 311 | MEF2C |
| 561 | 12 | 315 | HOXB13 |
| 562 | 12 | 343 | NR1H2 |
| 563 | 12 | 106 | HINFP |
| 564 | 12 | 325 | NR6A1 |
